# L-Theanine from *Bacillus tequilensis* metabolites inhibits *Alternaria alternata* via an *Nrf2*-dependent oxidative-stress and ABC-transporter regulatory axis

**DOI:** 10.3389/fpls.2026.1987466

**Published:** 2026-10-14

**Authors:** Qiuyan Bi, Fen Lu, Jie Wu, Xiangyu Liu, Jianjiang Zhao, Xiuying Han

**Affiliations:** Plant Protection Institute, Hebei Academy of Agriculture and Forestry Sciences, National Collection of Plant-associated Microbes (Hebei), IPM Innovation Center of Hebei, International Science and Technology Joint Research Center on IPM of Hebei, Baoding, China

**Keywords:** ABC transporter, *Alternaria alternata*, antifungal mechanism, bacillus tequilensis, L-Theanine, *Nrf2* transcription factor

## Abstract

**Background:**

Postharvest decay caused by *Alternaria alternata* and contamination with tenuazonic acid, alternariol, and tentoxin pose significant threats to pear fruit quality and safety. The reliance on synthetic fungicides is becoming unsustainable, and environmentally friendly alternatives are needed.

**Methods:**

L-Theanine was detected and quantified in crude metabolites of *Bacillus tequilensis* by LC-MS/MS using an authentic standard. Commercial L-theanine (purity ≥98%) was used to evaluate antifungal activity against *A. alternata*. The antifungal mechanism was investigated using transcriptomics, weighted gene co-expression network analysis (WGCNA), and targeted gene knockout. Antifungal assays used four technical replicates per concentration in two independent experiments. Transcriptomic and gene expression analyses used three biological replicates. Mutant phenotyping used three biological replicates with three technical replicates each.

**Results:**

L-Theanine inhibited mycelial growth (EC_50_ = 1.54 μg·mL^-1^; 95% CI 1.32–1.76 μg·mL^-1^) and spore germination (EC_50_ = 0.61 ± 0.05 μg·mL^-1^; 95% CI 0.52–0.70 μg·mL^-1^) and significantly reduced toxin production. Transcriptomic profiling revealed time-dependent downregulation of 12 ABC transporter genes, and WGCNA indicated Nrf2 as a candidate central node within the co-expression module. Functional validation revealed that *Nrf2* deletion markedly attenuated L-theanine susceptibility (EC_50_ increased 3.8-fold), abolished toxin suppression, and abrogated oxidative stress responses, including ROS accumulation, lipid peroxidation, and suppression of antioxidant enzyme activities. Complementation fully restored the wild-type phenotype. Mechanistically, L-theanine induced a transient transcriptional activation of antioxidant genes (*CAT*, *SOD*, *GPX*) in the wild-type strain, followed by a progressive decline in enzyme activities and subsequent oxidative damage, whereas the Δ*Nrf2* mutant failed to activate this transcriptional response. In detached pear fruit, injection of L-theanine into the same wound that received the pathogen significantly reduced lesion expansion. These results support a model in which Nrf2 is required for L-theanine-induced oxidative stress and for the suppression of toxin biosynthesis. Direct promoter binding of Nrf2 to ABC transporter genes was not demonstrated, and Nrf2-dependence of L-theanine-induced ABC transporter repression was not tested in this study.

**Conclusion:**

This study identifies Nrf2 as a pivotal molecular switch and establishes L-theanine as a promising candidate for further development in integrated management of postharvest pear diseases. The increased resistance of the Δ*Nrf2* mutant is attributable to the loss of the Nrf2-dependent oxidative-stress response rather than to changes in ABC transporter expression alone. Validation was performed under laboratory and detached-fruit conditions; storage, safety, stability, phytotoxicity, and fruit-quality studies are required before practical application.

## 1 Introduction

*Alternaria alternata*, which causes pear black spot (Song et al., 2025), is a major postharvest pathogen that leads to fruit decay (Sun et al., 2023) and produces toxins such as tenuazonic acid, alternariol, and tentoxin (Prencipe et al., 2023), all of which are serious food safety concerns. Currently, disease management predominantly depends on synthetic fungicides (Wisniewski et al., 2016); however, their extensive application has engendered issues related to resistance development, chemical residues, and environmental contamination, necessitating the exploration of environmentally sustainable, biobased alternatives (Islam et al., 2024).

*Bacillus tequilensis* produces a broad spectrum of antimicrobial metabolites (Xu et al., 2021), and members of the genus *Bacillus* are regarded as promising biocontrol agents against postharvest fungal diseases, mainly through antifungal lipopeptides and volatile metabolites (Wang et al., 2022). L-Theanine has been reported to inhibit phytopathogens by interfering with amino acid and carbohydrate metabolic pathways (Zhang et al., 2024), and omics-based approaches have begun to reveal the antifungal mechanisms of *Bacillus*-derived metabolites (Wang et al., 2021a). Nevertheless, whether *B. tequilensis* metabolites act directly on *A. alternata*, and which molecular targets mediate such activity, remain unknown.

ATP-binding cassette (ABC) transporters are central to fungal multidrug resistance, toxin secretion and detoxification, and members of the pleiotropic drug resistance (PDR) and multidrug resistance-associated protein (MRP) subfamilies are critical determinants of antifungal susceptibility (Khunweeraphong and Kuchler, 2021). In *Saccharomyces cerevisiae*, the transcription factor Yap1 regulates ABC transporter genes such as *YCF1* and *PDR5* (Wemmie et al., 1994), and Yap1 homologues in filamentous fungi modulate oxidative stress responses and secondary metabolism (Mendoza-Martínez et al., 2020). However, whether Nrf2 homologues regulate ABC transporter gene expression in plant pathogenic fungi is largely uncharacterized (Simaan et al., 2019). In *A. alternata*, the molecular targets of L-theanine and the regulatory networks through which it acts have not been defined. We therefore asked whether L-theanine acts through a transcription factor-mediated regulatory axis that couples ABC transporter expression to the oxidative stress response.

This study aimed to (i) detect and quantify L-theanine in *B. tequilensis* crude metabolites, (ii) evaluate its antifungal activity against *A. alternata in vitro* and on detached fruit, and (iii) test the hypothesis that L-theanine acts through an Nrf2-dependent regulatory axis influencing oxidative stress homeostasis, and (iv) examine the relationship between Nrf2 and ABC transporter expression, using transcriptomics, WGCNA, and targeted gene knockout. The gene we designated *Nrf2* (locus tag: AaNrf2_001; **Supplementary Table S9**) encodes a fungal transcription factor homologous to yeast Yap1/Nrf2-like oxidative-stress regulators.

## 2 Materials and methods

### 2.1 Strains, media, and reagents

*Bacillus tequilensis* strain 2_2a (maintained in our laboratory; accession number CGMCC No. 19446) and *Alternaria alternata* strain ACCC 36162 (China Agricultural Microbial Culture Collection Center) were used. Sigma-Aldrich supplied L-theanine (purity ≥98%), tentoxin (TEN), alternariol (ALT), and tenuazonic acid (TeA). TRIzol, PrimeScript™ RT reagent kit with gDNA Eraser, and SYBR^®^ Premix Ex Taq™ II were purchased from TaKaRa (Dalian, China). The WT and mutant strains of *A. alternata* are available from the corresponding author upon reasonable request.

### 2.2 Extraction of crude metabolites from *B. tequilensis* and detection and quantification of L-Theanine

*B. tequilensis* was cultured in Luria–Bertani (LB) medium at 30 °C with shaking at 180 rpm for 72 h. After centrifugation (10,000 × g, 15 min, 4 °C), the supernatant was extracted twice with an equal volume of ethyl acetate to remove lipophilic compounds; the aqueous phase was retained. L-Theanine was recovered from the aqueous phase by ultrasonication in deionized water at room temperature for 30 min, followed by centrifugation at 3000 × g for 10 min, according to the extraction principle of MOHWA0024.00 (Taiwan Food and Drug Administration, 2017), with modifications. The detailed parameters of any additional methanol-based recovery step were not fully recorded in the original laboratory records; therefore, only the steps that can be verified against the official method and the records are reported. The combined extract was evaporated to dryness and redissolved in dimethyl sulfoxide (DMSO) at a final concentration of 10 mg·mL^-1^.

LC-MS/MS analysis was performed on an Agilent 1260 HPLC coupled to a 6460 triple quadrupole mass spectrometer. Separation used an Agilent ZORBAX SB-Aq C18 column (2.1 × 100 mm, 1.8 μm) at 40 °C with a mobile phase of 0.1% formic acid in water (A) and methanol (B) at 0.3 mL·min^-1^. Gradient: 0–2 min 5% B, 2–10 min 5–95% B, 10–12 min 95% B, 12–15 min 5% B. Detection was in positive electrospray ionisation with multiple reaction monitoring for transitions *m/z* 175.1 → 46.2 and 175.1 → 84.1. The MRM transitions were optimized in our laboratory. Quantification was performed using an external calibration curve of authentic L-theanine standard. No isotopically labelled internal standard was used. The extraction and chromatographic conditions were adapted from MOHWA0024.00 (Taiwan Food and Drug Administration, 2017) and Hu et al. (2024). Bedner et al. (2010) is cited as a general methodological reference for LC-MS/MS determination of theanine. Data were acquired and processed with MassHunter software.

Identification of L-theanine was based on: (i) retention time matching with an authentic L-theanine standard (5.86 min); (ii) co-elution with the standard; (iii) the presence of two MRM transitions (*m*/*z* 175.1 → 46.2 and 175.1 → 84.1) with ion-ratio criteria within ±20% of the standard; and (iv) identical fragmentation patterns between the standard and the extract. Quantification was performed using an external calibration curve of L-theanine standard (0.1–500 ng·mL^-^¹; R² = 0.9992; **Supplementary Table S6**). Because the purpose of the LC-MS/MS analysis was to confirm the presence of L-theanine in the crude extract and to determine the concentration used for dosing, rather than to establish a fully validated quantitative method, LOD, LOQ, recovery, and precision were not determined. This is consistent with the fact that no preparative purification of L-theanine was performed and that commercial L-theanine standard (purity ≥98%) was used for all antifungal and mechanistic assays.

The concentration of L-theanine in the crude extract was independently quantified by LC-MS/MS against the external calibration curve (12.3 μg per mg crude extract; **Supplementary Table S6**), and all bioassays were dosed on the basis of this measured concentration rather than the nominal extract mass. Therefore, the extraction step does not affect the reported antifungal concentrations.

### 2.3 Evaluation of antifungal activity

#### 2.3.1 Inhibition of mycelial growth via the cup-plate method

*A. alternata* was cultured on potato dextrose agar (PDA) at 24 °C for seven days. Mycelial plugs (5 mm diameter) were punched from the colony edge and placed centrally on fresh PDA plates (90 mm diameter). Four wells (5 mm) were punched at 3 cm from the centre in a cross pattern. Each plate was used to test a single concentration with four replicate wells. To test six concentrations (0, 0.5, 1, 2, 4, 8 μg·mL^-1^), six separate plates were prepared per replicate. All plates were prepared with uniform medium thickness and mycelial plugs of consistent size from the same colony source. L-Theanine solutions (20 μL per well) were added, and plates were incubated at 24 °C until the control colony expanded to two-thirds of the plate diameter. Colony diameters were measured in two perpendicular directions, and inhibition rates were calculated as: Inhibition (%) = [(D_control – D_treatment)/(D_control – 5)] × 100, where D is colony diameter (mm) (Bi et al., 2023). Each concentration had four replicates (across wells). The experiment was independently repeated twice. Data from one representative experiment are shown; the independent repetition gave similar results. For mycelial growth inhibition, n = 2 independent biological replicates, each with four technical replicates.

#### 2.3.2 Spore germination inhibition

Spores were collected from 14-day-old PDA cultures and filtered through triple-layer gauze to remove mycelial debris. The spore suspension was adjusted to 1 × 10^4^ spores·mL^-1^. Water agar (WA) plates containing L-theanine at 0, 0.25, 0.5, 1, and 2 μg·mL^-1^ were prepared. Aliquots of 50 μL of the spore suspension were dispensed onto each plate, evenly distributed, and incubated at 24 °C in the dark for 8 hours. Spore germination was assessed under a light microscope using a 40× objective and a 10× ocular lens (total magnification 400×). A spore was considered germinated when the germ tube length exceeded the spore diameter (Uldahl and Knutsen, 2009). The experimental unit was the independent biological replicate. For each biological replicate, four microscopic fields and approximately 50 spores per field were examined. Germination percentages were calculated per biological replicate and used for statistical analysis (n = 3 independent biological replicates).

#### 2.3.3 Assessment of detached fruit lesions

Pear fruit (*Pyrus bretschneideri*) was surface-sterilized using 75% ethanol, rinsed with sterile water and air-dried. Three wounds (2 mm deep, 2 mm diameter) were made at the equator with a sterile cork borer. A 10 μL aliquot of L-theanine solution was injected into each wound to give a final concentration of 2 μg•mL^-^¹ in the wound; control wounds received 10 μL of sterile water. The solution remained within the wound without visible overflow. Immediately afterwards, a 5 mm diameter mycelial plug of *A. alternata* from a 7-day-old PDA culture was placed directly onto the wound surface, covering the wound, rather than being inserted into the 2 mm wound, to avoid displacement of the L-theanine solution. The plug was gently pressed to ensure stable contact with the wound surface. Because L-theanine was applied into the wound and the mycelial plug was placed directly onto the same wound surface, the treatment and the pathogen were co-localized at the same wound site, allowing direct assessment of the antifungal activity of L-theanine against *A. alternata* in planta. Fruits were placed in plastic boxes with moist filter paper to maintain 60–70% relative humidity and incubated at 24 °C under a 12 h light/dark photoperiod for 7 days. Lesion diameters were measured, and lesion areas were calculated as π × (diameter/2)² (Wang et al., 2024). Each treatment had 15 fruits, and the experiment was repeated twice.

### 2.4 Transcriptome sequencing and bioinformatic analysis

*A. alternata* was cultured in potato dextrose broth (PDB) at 24 °C with shaking at 120 rpm. L-Theanine was added to a final concentration of 2 μg·mL^-1^; the control received an equal volume of sterile water. Mycelia were harvested at 0, 6, 12, and 24 h post-treatment (three biological replicates per time point), flash-frozen in liquid nitrogen, and stored at –80 °C. Total RNA was extracted using TRIzol reagent (Chomczynski and Sacchi, 2006) with additional DNase I (Takara) treatment to remove genomic DNA. RNA quality was assessed on a 1% agarose gel and quantified with a NanoDrop 2000 (A_260_/A_280_ between 1.9 and 2.1). Libraries were prepared with the Illumina TruSeq RNA Sample Prep Kit and sequenced on an Illumina NovaSeq 6000 platform (150 bp paired-end reads). Raw reads were filtered with Trimmomatic v0.39 (parameters: LEADING:3, TRAILING:3, SLIDINGWINDOW:4:15, MINLEN:50) and aligned to the *A. alternata* reference genome (NCBI Assembly ASM306616v1) using HISAT2 v2.1.0 (Kim et al., 2015) with default parameters. Gene expression quantification was performed with featureCounts v2.0.0. Differential expression analysis was conducted with DESeq2 v1.30.1 (Love et al., 2014) using an FDR-adjusted *p* < 0.05 and |log_2_FC| ≥ 1 as thresholds. ABC transporter genes were identified based on the PFAM database entry PF00005 (Sonnhammer et al., 1997) and further confirmed by conserved-domain and phylogenetic analysis. Weighted gene co-expression network analysis (WGCNA) was performed using the WGCNA R package (Langfelder and Horvath, 2008) with a soft thresholding power of 6 (chosen to achieve scale-free topology). Modules were defined using the dynamic tree cut method with a minimum module size of 30 genes. Hub genes were identified based on module membership and gene significance. The detailed PCR conditions and thermal cycling parameters are provided in **Supplementary Table S2**. Detailed protocols for RNA extraction, cDNA synthesis, and RT-qPCR conditions are provided in **Supplementary Text S1**.

On the basis of the transcriptomic and WGCNA results, candidate hub genes were ranked by (i) module membership (kME) within the ABC transporter co-expression module, (ii) module connectivity, (iii) co-expression correlation with ABC transporter genes, and (iv) significance of differential expression after L-theanine treatment (|log_2_FC| ≥ 1, FDR < 0.05). *Nrf2* (locus tag AaNrf2_001) ranked highest among the transcription factors satisfying all four criteria, with a module connectivity of 0.82 and a co-expression correlation of *r* = 0.91 with ABC transporter genes (**Supplementary Table S10**). *Nrf2* was therefore selected as the primary candidate for functional validation by targeted gene knockout. Other candidate regulators (*AaCrz1*, *AaZ6C1*, *AaYap1-2*) were retained as secondary candidates for discussion.

### 2.5 Generation and characterisation of *Nrf2* knockout and complementation strains

To test the causal role of *Nrf2* in mediating the antifungal activity of L-theanine, we generated *Nrf2* deletion and complementation strains as described below.

#### 2.5.1 Construction of knockout vectors and protoplast transformation procedures

The *Nrf2* gene knockout construct was generated by the split marker PCR method (Catlett et al., 2003). The hygromycin B phosphotransferase (*hph*) gene, conferring resistance to hygromycin B, was used as a selectable marker. Upstream (1.2 kb) and downstream (1.2 kb) homology arms flanking the *Nrf2* open reading frame were amplified from genomic DNA of *A. alternata* using primers Nrf2 up F/R and Nrf2 down F/R (**Supplementary Table S1**). The *hph* cassette (1.0 kb) was amplified from pCB1004 using primers HPH F and HPH R. Fusion PCR was performed to join the upstream arm to the 5′ end of *hph* and the downstream arm to the 3′ end of *hph*, yielding two overlapping fragments. Protoplasts were prepared from young mycelia (16 h culture in PDB) by enzymatic digestion in 0.7 M NaCl containing 10 mg·mL^-1^ driselase, 5 mg·mL^-1^ snailase, and 5 mg·mL^-1^ lywallzyme at 28 °C for 4 h with gentle shaking (Qi et al., 2025). Protoplasts were filtered through Miracloth, washed twice with STC buffer (1 M sorbitol, 50 mM Tris-HCl pH 8.0, 50 mM CaCl_2_), and resuspended at 10^8^ protoplasts·mL^-1^. For transformation, 100 μL of protoplast suspension (~10^7^) was mixed with 10 μg of each fusion PCR fragment in the presence of 40% PEG 4000 (in STC buffer) and incubated on ice for 30 min, then at room temperature for 20 min. The mixture was placed on regeneration medium (PDA with 0.7 M sucrose) containing 200 μg·mL^-1^ hygromycin B and incubated at 24 °C for 7 days. Putative transformants were subcultured on fresh selective medium. The fusion PCR conditions are detailed in **Supplementary Table S2**.

#### 2.5.2 Molecular confirmation of gene deletion mutants

Genomic DNA was extracted from mycelia using the CTAB method. PCR verification used the following primer sets: (i) internal gene primers F1 and R1 (amplifying a 1.5 kb fragment from the WT *Nrf2* locus), (ii) *hph*-specific primers HPH F/R (1.0 kb product), and (iii) flanking primers Up F and Down R (expected 1.3 kb for WT, 1.7 kb for KO due to insertion of *hph* cassette). For Southern blot analysis, 20 μg of genomic DNA was digested with *EcoRV* (which cuts within the *Nrf2* locus but not in the *hph* cassette), electrophoresed on a 0.8% agarose gel, and transferred to a positively charged nylon membrane (Hybond N+, GE Healthcare). A DIG-labelled 500 bp probe complementary to the upstream flanking region was generated by PCR using DIG-11-dUTP (Roche) with primers Probe F and Probe R. Hybridisation and detection were performed according to the DIG Application Manual (Roche). A second blot was probed with a DIG-labelled *hph* fragment to confirm integration (Gold et al., 2000). The PCR programs for verification are provided in **Supplementary Table S2**. Buffer compositions for Southern blot analysis are provided in **Supplementary Table S3**. Detailed procedures for DNA digestion, gel electrophoresis, capillary transfer, probe hybridization, stringency washes, and chemiluminescent detection are described in **Supplementary Text S3**.

#### 2.5.3 Development of complementation strains

For complementation, the native *Nrf2* gene including its promoter (1.5 kb upstream) and terminator (0.5 kb downstream) was amplified and cloned into the pG418 vector (containing the G418 resistance cassette) to produce pG418-*Nrf2*-native. The construct was confirmed by sequencing and then introduced into Δ*Nrf2* protoplasts using the same PEG-mediated transformation method. Transformants were selected on regeneration medium containing 200 μg·mL^-1^ G418 and confirmed by PCR and RT-qPCR for restoration of *Nrf2* expression (Wang et al., 2021b).

#### 2.5.4 Phenotypic characterization of the mutant strains

##### 2.5.4.1 Sensitivity to L-Theanine

The EC_50_ values for mycelial growth inhibition were determined for WT, Δ*Nrf2*, and Δ*Nrf2*-C strains using the cup-plate method described in section 2.3.1, with L-theanine concentrations ranging from 0 to 8 μg·mL^-1^. Dose–response curves were fitted by four-parameter logistic regression in GraphPad Prism 8.0. EC_50_ values are reported with 95% confidence intervals and R². Three independent biological replicates were performed, each with three technical replicates.

##### 2.5.4.2 Toxin production and gene expression

For toxin accumulation, strains were cultured in PD broth with or without 2 μg·mL^-1^ L-theanine for 10 days at 24 °C, 120 rpm. The 10-day time point was selected based on preliminary experiments showing that toxin accumulation reached a stable plateau by day 10. The culture filtrate was extracted with an equal volume of ethyl acetate, and the extract was evaporated and reconstituted in methanol. Quantification of TEN, ALT, and TeA was performed by LC-MS/MS (De Berardis et al., 2018) using a Waters ACQUITY UPLC BEH C18 column (2.1 × 100 mm, 1.7 μm) with a gradient of water (0.1% formic acid) and acetonitrile. External standard curves (0.1–500 ng·mL^-1^) were used for quantification. Toxin amounts were normalized to mycelial dry weight. For gene expression analysis, mycelia were harvested at 12 h post-treatment, based on preliminary time-course data showing peak differential expression of toxin biosynthesis genes at this time point; RNA was extracted, and RT-qPCR was performed for *TES*, *PksA*, and *PksJ* using specific primers (Liu et al., 2020). *β*-Tubulin served as the reference. Relative expression was calculated by the 2^-^ΔΔCt method (Livak and Schmittgen, 2001). The RT-qPCR cycling conditions are shown in **Supplementary Table S2**. Three independent biological replicates were performed, each with three technical replicates.

##### 2.5.4.3 Oxidative stress biomarkers

Mycelia were grown in PD broth with 2 μg·mL^-1^ L-theanine for 48 h. Intracellular ROS was detected by incubating mycelia with 10 μM DCFH-DA (Beyotime) at 37 °C for 1 h, followed by washing and observation under a fluorescence microscope (excitation 485 nm, emission 528 nm). Fluorescence intensity was quantified using ImageJ software (mean fluorescence intensity per unit area) and is reported as relative fluorescence units (RFU) (**Supplementary Figure S3**). For biochemical assays, mycelia were ground in liquid nitrogen and homogenised in cold PBS. MDA content was determined using the TBA method (Hodges et al., 1999): 0.5 mL of supernatant was mixed with 1 mL of 0.5% TBA in 20% TCA, boiled for 30 min, centrifuged, and absorbance read at 532 and 600 nm. SOD activity was assayed by NBT photoreduction (Beauchamp and Fridovich, 1971): one unit of SOD was defined as the amount causing 50% inhibition of NBT reduction. CAT activity was measured by following the decrease in H_2_O_2_ absorbance at 240 nm (Beers and Sizer, 1952). GSH content was determined by the DTNB cycling method (Tietze, 1969) using a standard curve. GST activity was measured with 1-chloro-2, 4-dinitrobenzene (CDNB) as substrate (Habig et al., 1974); one unit was defined as the amount catalysing the formation of 1 μmol of GSH-CDNB conjugate per minute at 30 °C, pH 6.5. The detailed reaction conditions, reagent concentrations, and calculation formulas for MDA, SOD, CAT, GSH, and GST assays are provided in **Supplementary Text S2**. Three independent biological replicates were performed, each with three technical replicates.

##### 2.5.4.4 Antioxidant gene expression

RT-qPCR for *AaCAT*, *AaSOD*, and *AaGPX* was performed on RNA extracted from mycelia treated with 2 μg·mL^-1^ L-theanine for 0, 1, 3, 6, and 12 h. Primer sequences are given in **Supplementary Table S1**. Relative expression was normalised to *β*-tubulin. The thermal cycling protocol is given in **Supplementary Table S2**. Three independent biological replicates were performed, each with three technical replicates.

### 2.6 Statistical analysis

All data are presented as means ± standard deviation (SD) of three independent biological replicates (each with three technical replicates) unless otherwise stated. For mycelial growth inhibition assays, each concentration included four technical replicates per independent experiment, and the experiment was independently repeated twice (n = 2 independent biological replicates, each with four technical replicates). For spore germination assays, the experimental unit was the independent biological replicate (n = 3), with four microscopic fields and approximately 50 spores per field examined per replicate. Statistical analyses were performed using SPSS 22.0. For comparisons involving multiple groups and time points, two-way ANOVA followed by Tukey’s post-hoc test was used. For two-group comparisons, Student’s t-test (two-tailed) was applied; paired t-tests were used when the same samples were compared across treatments (e.g., **Tables 1**, **2**, **3**), and unpaired t-tests were used when independent groups were compared (e.g., **Table 4**). A *p*-value < 0.05 was considered significant. EC_50_ values were calculated by fitting a four-parameter logistic model in GraphPad Prism 8.0, and 95% confidence intervals and goodness-of-fit (R²) is reported. Detailed statistical formulas, including the four-parameter logistic model for EC_50_ calculation, and the raw RNA-seq data deposition information are provided in **Supplementary Text S4**.

**Table 1:** RT-qPCR validation of ABC transporter genes^a^.

Gene ^b^	RNA-Seq (log_2_FC) ^c^	qRT-PCR (log_2_FC) c	*p*-value d
*AaABC1*	–4.50± 0.40	–4.15 ± 0.45	<0.001
*AaABC-G1*	–1.72± 0.35	–2.05 ± 0.32	<0.01
*AaABC-G2*	–1.11± 0.19	–1.39 ± 0.28	<0.05
*AaABC-G3*	–1.66± 0.22	–1.50 ± 0.19	<0.05
*AaPDR1*	–2.84± 0.25	–2.06 ± 0.47	<0.01
*AaABC-C1*	–1.19± 0.17	–1.32 ± 0.24	<0.05
*AaMRP2*	–1.69± 0.12	–1.51 ± 0.16	<0.05
*AaABC2*	–3.20± 0.35	–2.78 ± 0.36	<0.001
*AaOPT3*	–2.15± 0.05	–2.10 ± 0.10	<0.01
*AaATM1*	–1.98± 0.11	–1.80 ± 0.14	<0.01
*AaMDR1*	–1.52± 0.15	–1.46 ± 0.14	<0.05
*AaABC-B2*	–0.98± 0.12	–1.03 ± 0.22	<0.05

^a^CK, control group; T, L-theanine-treated group (2 μg·mL^-1^). Samples collected at 12 h. RNA-Seq data from **Table 6**; qRT-PCR values are mean ± SD (n = 3 independent biological replicates, each with three technical replicates). ^b^ Gene names italicized. ^c^ Negative log_2_FC indicates downregulation. d *p*-values from paired t-test vs control; all genes significant (*p* < 0.05).

**Table 2:** RT-qPCR validation of transcription factor genes^a^.

TF ID	Gene name ^b^	Domain	RNA-Seq (log_2_FC) ^c^	qRT-PCR (log_2_FC) ^c^	*p*-value ^d^
NRF2	*Nrf2*	bZIP	–2.35 ± 0.24	–2.18 ± 0.28	<0.001
AACRZ1	*AaCrz1*	C2H2	–1.68 ± 0.18	–1.55 ± 0.22	<0.01
AABC-C1-TF	*AaZ6C1*	Zn_2_Cys_6_	–2.12 ± 0.21	–1.98 ± 0.26	<0.01
AaYap1	*AaYap1-2*	bZIP	+1.42 ± 0.12	+1.35 ± 0.18	<0.01

^a^CK, control group; T, L-theanine-treated group (2 μg·mL^-1^). Samples collected at 12 h. RNA-Seq data from **Supplementary Table S10**; qRT-PCR values are mean ± SD (n = 3 independent biological replicates, each with three technical replicates). ^b^Gene names italicized. ^c^Negative log_2_FC = downregulation; positive log_2_FC = upregulation. ^d^*p*-values from paired t-test vs control; all genes significant (*p* < 0.05).

**Table 3:** Effects of oxidative stress caused by L-Theanine on different *A. alternata* strains.

Strain	Treatment^a^	ROS (RFU) ^b,c^	MDA (nmol·g^-^¹ protein) ^c^	SOD (U·mg^-1^ protein) ^c^	CAT (U·mg^-1^ protein) ^c^
WT	–	1.00 ± 0.08b	2.5 ± 0.3b	50.2 ± 4.1a	15.6 ± 1.8a
WT	+	4.21 ± 0.45a	12.1 ± 1.1a	42.3 ± 3.9b	8.7 ± 1.2b
Δ*Nrf2*	–	0.98 ± 0.07b	2.4 ± 0.3b	51.0 ± 2.8a	15.8 ± 1.4a
Δ*Nrf2*	+	1.12 ± 0.10b	3.8 ± 0.6b	49.9 ± 3.6a	14.7 ± 1.0a
Δ*Nrf2*-C	–	0.97 ± 0.06b	2.2 ± 0.1b	52.6 ± 3.4a	16.0 ± 1.9a
Δ*Nrf2*-C	+	4.02 ± 0.38a	11.7 ± 1.4a	45.3 ± 4.0b	9.1 ± 1.0b

^a^“–” denotes untreated control (without L-theanine); “+” denotes treatment with L-theanine at 2 μg·mL^-^¹ for 48 h. ^b^ROS quantified as relative fluorescence units (RFU) normalized to untreated WT control (set to 1.00). ^c^Values are mean ± SD (n = 3 independent biological replicates, each with three technical replicates). *p* < 0.05 vs the untreated control of the same strain (paired t-test).

**Table 4:** Relative expression of antioxidant enzyme genes in WT and Δ*Nrf2* treated with L-Theanine^a^.

Gene	Strain	0 h ^b^	1 h ^b^	3 h ^b^	6 h ^b^	12 h ^b^
*CAT*	WT	1.00 ± 0.05 a	3.50 ± 0.61 a	8.21 ± 1.07 a	5.10 ± 0.82 a	2.12 ± 0.34 a
*CAT*	Δ*Nrf2*	1.00 ± 0.08 a	1.21 ± 0.22 b	1.84 ± 0.39 b	1.48 ± 0.31 b	1.14 ± 0.21 b
*SOD*	WT	1.00 ± 0.06 a	4.11 ± 0.70 a	10.42 ± 1.52 a	6.19 ± 0.92 a	2.81 ± 0.44 a
*SOD*	Δ*Nrf2*	1.00 ± 0.10 a	1.54 ± 0.34 b	2.50 ± 0.54 b	2.00 ± 0.37 b	1.40 ± 0.20 b
*GPX*	WT	1.00 ± 0.04 a	2.83 ± 0.48 a	5.51 ± 0.76 a	3.81 ± 0.60 a	1.88 ± 0.32 a
*GPX*	Δ*Nrf2*	1.00 ± 0.07 a	1.06 ± 0.23 b	1.54 ± 0.28 b	1.32 ± 0.22 b	1.02 ± 0.11 b

^a^L-theanine concentration 2 μg·mL^-^¹. ^b^Values are mean ± SD (n = 3 independent biological replicates, each with three technical replicates) and were normalized to 0 h for the same strain (set to 1.00). Gene names are italicized. Different lowercase letters at the same time point indicate significant differences between WT and Δ*Nrf2* (*p* < 0.05, unpaired t-test). WT expression showed a transient upregulation peak at 3 h, whereas Δ*Nrf2* expression showed significantly weaker induction (1.8-, 2.5-, and 1.5-fold, respectively), confirming that Nrf2 is required for the full transcriptional activation of antioxidant defenses.

## 3 Results

### 3.1 Detection and Quantification of L-Theanine and its antifungal activity

LC-MS analysis of the *B. tequilensis* crude extract revealed a prominent peak at a retention time of 5.86 min, which co-eluted with the L-theanine standard (**Supplementary Figure S1A**). Both the standard and the extract showed identical fragmentation patterns with characteristic ions at *m/z* 46.2 and 84.1 (**Supplementary Figures S1B, C**), confirming the presence of L-theanine. Quantitative analysis indicated that L-theanine was present at a concentration of 12.3 μg per mg of crude extract (**Supplementary Table S6**), supporting its description as a major metabolite. No preparative purification of L-theanine from the crude extract was performed; commercial L-theanine standard (purity ≥98%) was used for all subsequent antifungal assays.

L-Theanine concentration-dependently inhibited the mycelial growth of *Alternaria alternata* (**Figures 1A–G**). The complete dose–response data, including inhibition rates at each concentration and the number of independent biological replicates, are provided in **Supplementary Table S7**. At concentrations of 0.5, 1, 2, 4, and 8 μg·mL^-1^, the inhibition rates were 25.2% ± 2.1%, 43.8% ± 3.5%, 78.5% ± 4.2%, 89.1% ± 2.7%, and 96.4% ± 1.5%, respectively (n = 2 independent biological replicates, each with four technical replicates). The EC_50_ for mycelial growth inhibition was 1.54 ± 0.12 μg·mL^-1^ (95% CI: 1.32–1.76 μg·mL^-1^; R² = 0.98; Hill slope = –1.24 ± 0.15), consistent with the values obtained in subsequent experiments (**Table 5**; **Supplementary Table S7**). The dose-response curve with the four-parameter logistic fit is provided in **Supplementary Figure S8**.

**Figure 1:**
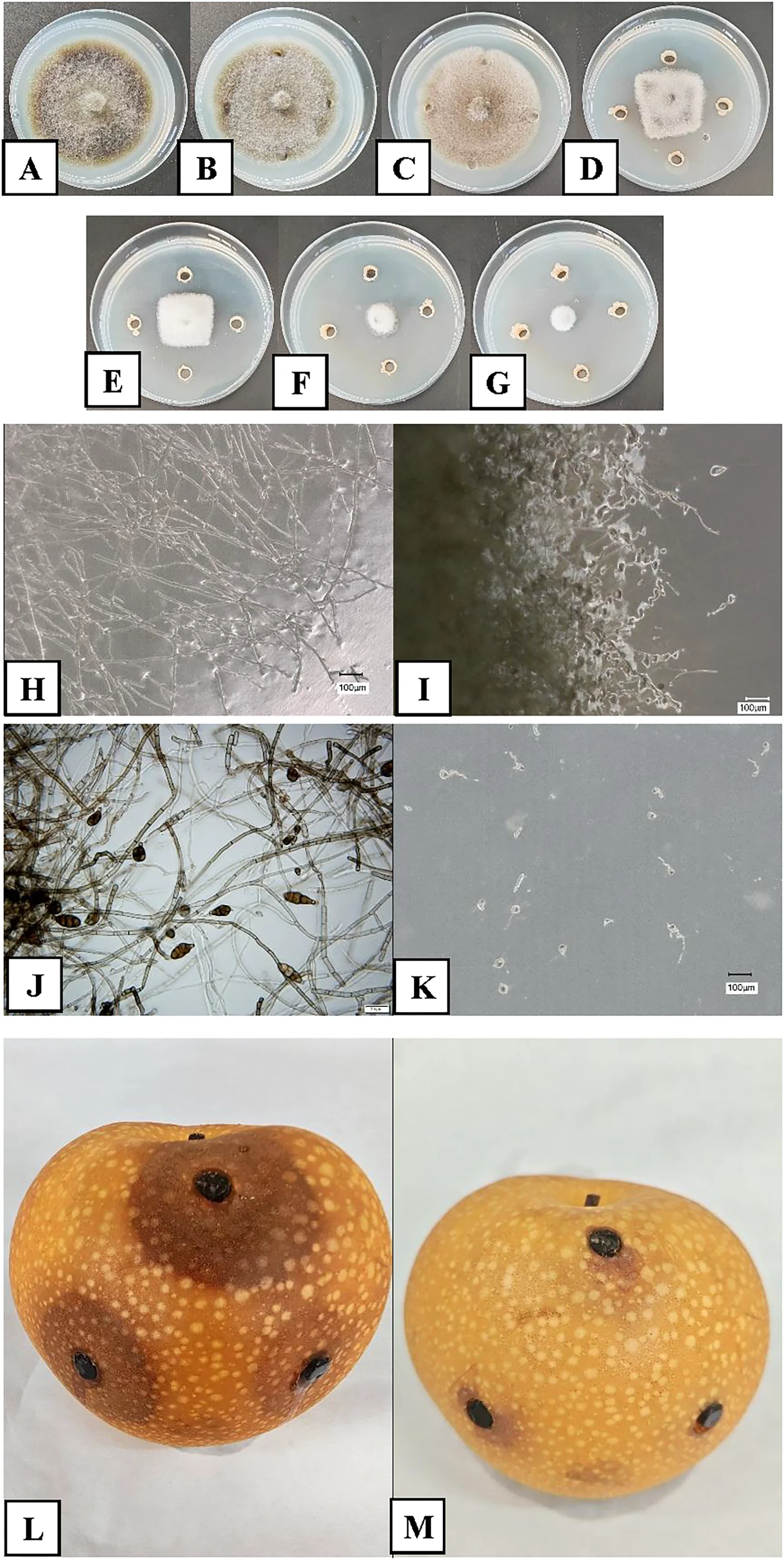
Concentration-dependent antifungal effects of L-theanine on *A. alternata*. **(A)** Blank control (no treatment). **(B)** Water control. **(C)** 0.5 μg·mL^-1^. **(D)** 1 μg·mL^-1^. **(E)** 2 μg·mL^-1^. **(F)** 4 μg·mL^-1^. **(G)** 8 μg·mL^-1^. **(H)** Mycelial morphology of control (200×). **(I)** Treated mycelia showing distortion, shrinkage, and vacuolation (200×). **(J)** Spore germination in control (400×). **(K)** Spores treated with 1 μg·mL^-1^ L-theanine, showing swollen germ tubes and delayed germination (400×). **(L)** Lesion development on detached pear fruit (control). **(M)** Lesion on fruit treated with 2 μg·mL^-1^ L-theanine injected into the wound, with the mycelial plug placed onto the wound surface, showing reduced lesion expansion.

**Table 5:** Sensitivity of Δ*Nrf2* to L-Theanine^a^.

Strain	EC_50_ (μg·mL^-1^) ^b^	95% CI (μg·mL^-1^) ^c^	R² ^d^
WT	1.54 ± 0.12 b	1.32–1.76	0.98
Δ*Nrf2*	5.80 ± 0.59 a	5.02–6.58	0.97
Δ*Nrf2*-C	1.42 ± 0.11 b	1.21–1.63	0.99

^a^EC_50_ determined by mycelial growth inhibition (0-8 μg·mL^-1^). ^b^ Values are mean ± SD (n = 3 independent biological replicates, each with four technical replicates). ^c^ 95% confidence interval from four-parameter logistic regression. ^d^ Goodness-of-fit (R²) for the fitted dose–response curve. Different lowercase letters indicate significant differences among strains (*p* < 0.05, one-way ANOVA with Tukey’s test).

For spore germination, L-theanine also showed concentration-dependent inhibition. The complete dose–response data are provided in **Supplementary Table S7**. At concentrations of 0.25, 0.5, 1, and 2 μg·mL^-^¹, the inhibition rates were 28.6% ± 2.4%, 46.2% ± 3.1%, 74.8% ± 3.8%, and 92.3% ± 2.6%, respectively (n = 3 independent biological replicates). The EC_50_ value for spore germination inhibition was determined to be 0.61 ± 0.05 μg·mL^-1^ (95% CI: 0.52–0.70 μg·mL^-1^; R² = 0.97; Hill slope = –1.18 ± 0.12). The experimental unit was the independent biological replicate; for each replicate, four microscopic fields and approximately 50 spores per field were examined.

Microscopic observation showed that treated mycelia became distorted, shrunken, and vacuolated (**Figure 1I**) compared with the uniform, smooth control mycelia (**Figure 1H**). Spores treated with L-theanine exhibited delayed germination, with swollen, abnormal germ tubes (**Figure 1K**) as opposed to the normal elongated germ tubes in the control (**Figure 1J**). In assays using detached pear fruit, injection of 2 μg·mL^-1^ L-theanine into the same wound that received the pathogen reduced lesion expansion by 76.0% ± 1.9% compared with the control (**Figures 1L, M**).

### 3.2 Transcriptomic profiles and ABC transporter downregulation

RNA-seq data were of high quality, with Q30 scores above 93% and alignment rates exceeding 94% for all samples (**Supplementary Table S4**). Principal component analysis (PCA) showed a clear separation between control and L-theanine-treated samples along PC1 (explaining 78% of variance; **Figure 2A**). At 12 h post-treatment, a total of 1,872 genes were differentially expressed (|log_2_FC| ≥ 1, FDR < 0.05), with 1,023 upregulated and 849 downregulated (**Figure 2B**). Among the ABC transporter gene family, 13, 15, and 12 genes showed differential expression at 6, 12, and 24 h, respectively, with the majority being downregulated (**Supplementary Table S5**).

**Figure 2:**
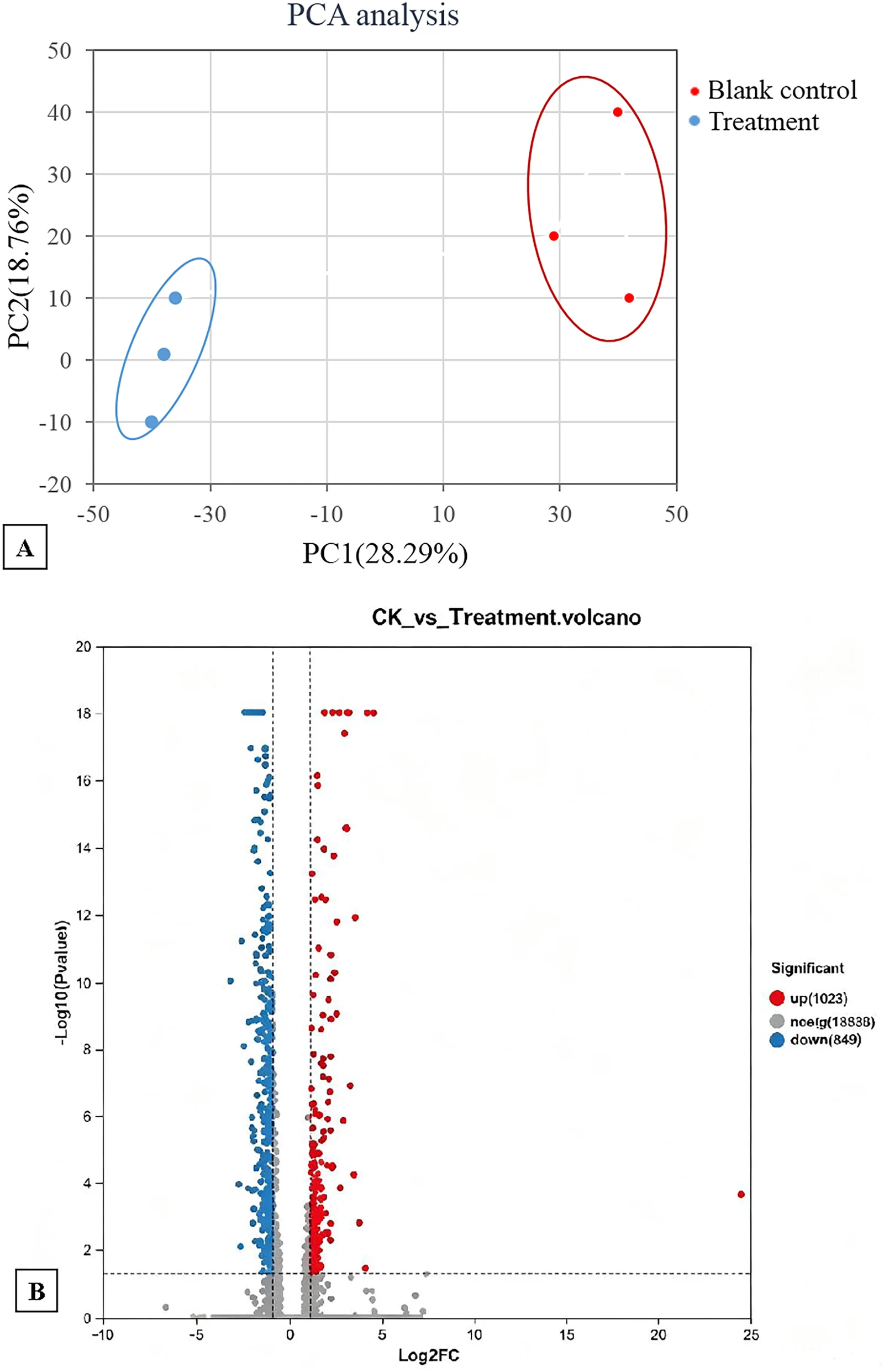
Transcriptomic analysis of *A. alternata* treated with L-theanine (2 μg·mL^-1^) for 12 h. **(A)** Principal component analysis (PCA) of control (CK) and treated (T) samples (n = 3 each). **(B)** Volcano plot of differentially expressed genes (|log_2_FC| ≥ 1, adjusted *p* < 0.05). Red: upregulated; blue: downregulated; grey: not significant.

In the *A. alternata* genome, we identified 42 ABC transporter genes based on PFAM and phylogenetic analysis. Among these, 12 genes were significantly downregulated at 12 h (**Table 6**). The most strongly repressed was *AaABC1* (a homologue of yeast *PDR5*), with a log_2_FC of –4.50, followed by *AaABC2* (–3.20), *AaPDR1* (–2.84), *AaOPT3* (–2.15), *AaATM1* (–1.98), and others.

**Table 6:** Differentially expressed ABC transporter genes in *A. alternata*^a^.

Gene ID ^b^	Annotation	Log_2_FC	Direction	Predicted functional association ^c^
*AaABC1*	Yeast PDR5 homologue	–4.50	Down	Efflux of mycotoxins and antimicrobials
*AaABC-G1*	PDR family	–1.72	Down	Efflux of toxic substances
*AaABC-G2*	PDR family	–1.11	Down	Possibly involved in secondary metabolism
*AaABC-G3*	PDR family	–1.66	Down	Toxin biosynthesis pathway (predicted)
*AaPDR1*	PDR-type MDR transporter	–2.84	Down	Stress response, toxin efflux
*AaABC-C1*	MRP family	–1.19	Down	Glutathione conjugate transport (predicted)
*AaMRP2*	MRP-type	–1.69	Down	Glutathione conjugate transport (predicted)
*AaABC2*	Oligopeptide transporter homologue	–3.20	Down	Nutrient uptake
*AaOPT3*	Oligopeptide transporter	–2.15	Down	Nutrient absorption
*AaATM1*	Mitochondrial ABC transporter	–1.98	Down	Iron-sulfur cluster transport
*AaMDR1*	Mitochondrial ABC-B family	–1.52	Down	Drug efflux
*AaABC-B2*	Mitochondrial ABC	–1.02	Down	Mitochondrial peptide export

^a^CK, control group; T, L-theanine-treated group (2 μg·mL^-1^). Samples collected at 12 h. ^b^ Gene names are italicized. ^c^ Functional annotations from KEGG/Pfam/literature; functions preceded by “predicted” are inferred from homologues rather than experimentally demonstrated in *A. alternata*.

Gene Ontology (GO) analysis of the differentially expressed ABC transporter genes revealed significant enrichment in categories related to transmembrane (GO:0055085), drug (GO:0006855) transport, toxin efflux (GO:1990961), response to stimuli (GO:0042221), transcriptional regulation (GO:0006355), ATP binding (GO:0005524), ABC transporter activity (GO:0042626), DNA-binding transcription factor activity (GO:0003700), and membrane components (GO:0005886, GO:0016021) (**Figure 3A**).

**Figure 3:**
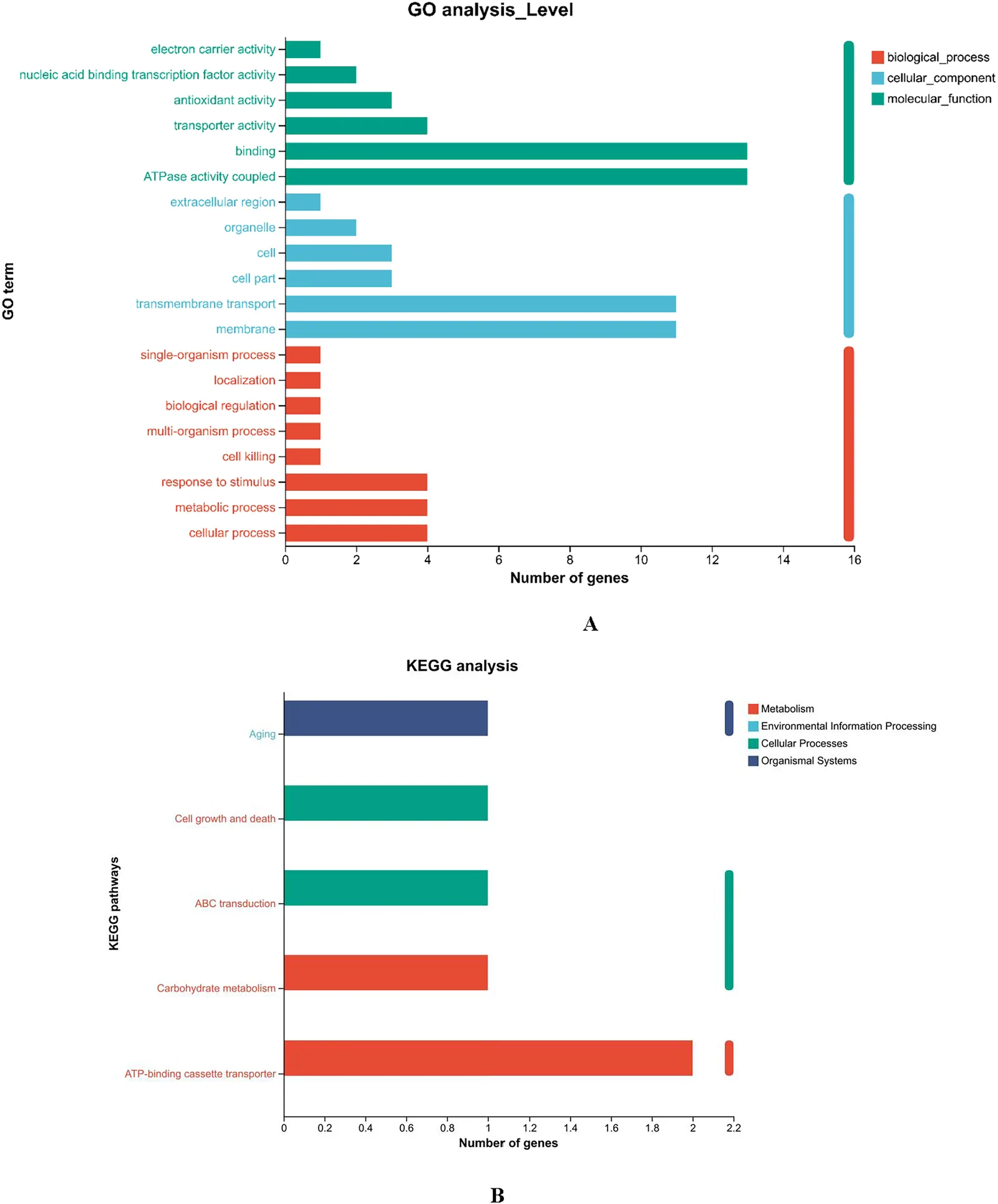
Enrichment analysis of differentially expressed genes. **(A)** Top GO terms in biological process (BP), cellular component (CC), and molecular function (MF). **(B)** KEGG pathway enrichment; dot size indicates gene count, colour indicates adjusted *p*-value.

Kyoto Encyclopedia of Genes and Genomes (KEGG) pathway enrichment analysis (**Table 7**) revealed that the differentially expressed genes (DEGs) were predominantly associated with the ABC transporter pathway (ko02010), whose expression was significantly enriched (*p* = 3.2 × 10^-6^). Additional enrichment was noted in pathways related to secondary metabolite biosynthesis (ko01110) and fungal drug resistance mechanisms, including the MAPK signaling pathway (ko04011) (**Figure 3B**).

**Table 7:** KEGG pathway enrichment of differentially expressed genes^a^.

Pathway	Fold enrichment ^b^	FDR ^c^	DEGs involved	Background genes ^d^
ABC transporters	2.64	0.0001	15	42
Drug transmembrane transport	2.31	0.0003	35	112
Transmembrane transport	1.92	0.0012	128	493
Calcium signalling pathway	1.76	0.0025	56	235
MAPK signalling pathway	1.58	0.0087	48	224

^a^DEGs were defined as those whose |log_2_FC| ≥ 1 and adjusted *p* < 0.05; L-theanine treated group (2 μg·mL^-1^) vs control at 12 h. ^b^ Fold enrichment is normalized against background gene distribution. ^c^ FDR by Benjamini–Hochberg. ^d^ Background: All protein-coding genes annotated in the *A. alternata* KEGG database.

### 3.3 WGCNA identifies *Nrf2* as a candidate central node

Although the WGCNA analysis is constrained by the limited number of time points and should be considered exploratory, the high connectivity and co-expression correlation consistently implicate Nrf2 as a key node in this network. Weighted gene co-expression network analysis (WGCNA) indicated Nrf2 as a candidate central node within the ABC transporter co-expression module, which exhibited high module connectivity (0.82) and a strong co-expression correlation with ABC transporter genes (*r* = 0.91) (**Supplementary Table S10**). The topological overlap matrix (TOM) heatmap and module-trait correlation heatmap are provided in **Supplementary Figure S2**. Other transcription factors, such as AaCrz1 (C2H2, connectivity 0.78) and AaSln1 (connectivity 0.65), also showed moderate co-expression. The expression of *Nrf2* itself was significantly downregulated at 12 h (log_2_FC = –2.35, FDR = 0.0003; **Table 8**). Several other candidate regulators, including *AaCrz1* (–1.68), *AaZ6C1* (–2.12), and *AaYap1-2* (+1.42), were also differentially expressed, suggesting a complex regulatory network.

**Table 8:** Candidate transcription factors associated with ABC regulation (12 h)^a^.

TF ID	Gene name ^b^	Domain	Log_2_FC ^c^	FDR ^d^	Regulation ^e^
NRF2	*Nrf2*	bZIP	–2.35	0.0003	Significantly down
AACRZ1	*AaCrz1*	C2H2	–1.68	0.0012	Significantly down
ABCAAYAP1	*AaYap1-2*	bZIP	+1.42	0.0085	Significantly up
AABC-C1-TF	*AaZ6C1*	Zn_2_Cys_6_	–2.12	0.0007	Significantly down

^a^CK, control group; T, L-theanine-treated group (2 μg·mL^-1^). Candidate TFs were selected by WGCNA and co-expressed with ABC genes (**Supplementary Table S10**). ^b^ Gene names italicized. ^c^ log_2_FC: + up, – down. ^d^ FDR by Benjamini–Hochberg. ^e^ Significant: |log_2_FC| ≥ 1 and FDR < 0.05.

Validation of the RNA-seq data by RT-qPCR for 12 ABC transporter genes (**Table 1**) and 4 transcription factor genes (**Table 2**) showed strong concordance, with correlation coefficients (R²) exceeding 0.9 for all tested genes, confirming the reliability of the transcriptomic data.

### 3.4 Functional validation of the critical role of *Nrf2* in mediating L-Theanine effects through gene knockout studies

The Δ*Nrf2* mutant was successfully generated and confirmed by multiple lines of evidence. PCR with internal primers (F1/R1) amplified a 1.5 kb fragment in the wild type (WT) but not in the knockout (KO) strain, while *hph*-specific primers amplified a 1.0 kb band only in the KO (**Figure 4A**). Flanking primers (Up F/Down R) gave a 1.3 kb product for WT and a 1.7 kb product for KO, consistent with the insertion of the *hph* cassette. Southern blot analysis (**Figure 4B**) using a flanking probe showed a 5.2 kb band in WT and a 4.8 kb band in KO, confirming the replacement of the *Nrf2* locus. The *hph* probe hybridised only to the KO DNA, giving a 4.8 kb band, confirming single-copy integration.

**Figure 4:**
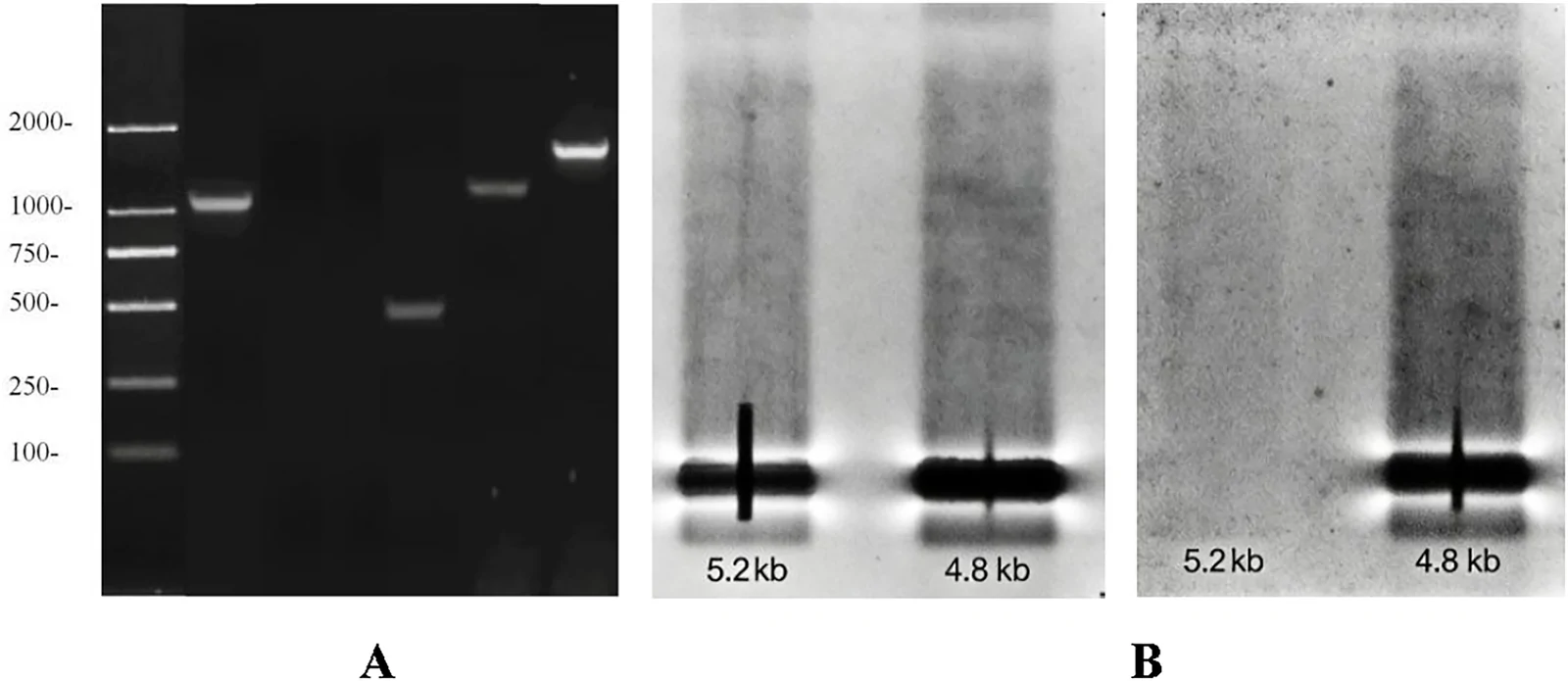
Confirmation of *Nrf2* knockout mutants. **(A)** PCR verification: M, DNA marker; Lane 1, WT with internal primers (F1/R1, 1.5 kb); Lane 2, KO with internal primers (no band); Lane 3, WT with *hph* primers (no band); Lane 4, KO with *hph* primers (1.0 kb); Lane 5, WT with flanking primers (Up F/Down R, 1.3 kb); Lane 6, KO with flanking primers (1.7 kb). **(B)** Southern blot: left, hybridisation with flanking probe (WT: 5.2 kb; KO: 4.8 kb); right, hybridisation with *hph* probe (KO only, 4.8 kb).

RT qPCR analysis of basal ABC transporter gene expression in untreated mycelia (**Table 9**) revealed that the transcript levels of *AaABC1*, *AaABC2*, *AaABC-C1*, *AaABC-G2*, *AaOPT3*, *AaYAP1*, and *AaMDR1* were significantly reduced to 28–45% of WT levels in the Δ*Nrf2* mutant. In contrast, *AaPDR1* expression was not significantly changed. Complementation (Δ*Nrf2*-C) restored the expression of these genes to near WT levels. These data describe basal expression only; they do not test whether L-theanine-induced repression of these genes requires Nrf2.

**Table 9:** Relative expression of ABC transporter genes in Δ*Nrf2*.

Gene	WT^a^	Δ*Nrf2*^a^	Δ*Nrf2*-C^a^	Trend ^b^
*AaABC1*	1.00 ± 0.08	0.30 ± 0.04*	0.90 ± 0.08	Significantly down
*AaABC2*	1.00 ± 0.09	0.34 ± 0.03*	0.94 ± 0.10	Significantly down
*AaABC-C1*	1.00 ± 0.09	0.28 ± 0.04*	0.92 ± 0.10	Significantly down
*AaPDR1*	1.00 ± 0.12	0.91 ± 0.08	1.05 ± 0.11	No change
*AaABC-G2*	1.00 ± 0.10	0.45 ± 0.06*	0.88 ± 0.09	Significantly down
*AaOPT3*	1.00 ± 0.09	0.35 ± 0.06*	0.89 ± 0.12	Significantly down
*AaYAP1*	1.00 ± 0.11	0.41 ± 0.05*	0.90 ± 0.11	Significantly down
*AaMDR1*	1.00 ± 0.11	0.32 ± 0.05*	0.89 ± 0.09	Significantly down

^a^Values are mean ± SD (n = 3 independent biological replicates, each with three technical replicates). ^b^ “Significantly down” indicates a statistically significant decrease in expression compared with that in the WT group (*p* < 0.05); “No change” indicates no significant difference. *p* < 0.05 vs WT (Student’s t-test, two-tailed). Complementation restored expression to near-WT levels, confirming knockout specificity.

Phenotypically, the Δ*Nrf2* mutant showed markedly reduced sensitivity to L-theanine. The EC_50_ for mycelial growth inhibition increased from 1.54 ± 0.12 μg·mL^-1^ (95% CI: 1.32–1.76) in WT to 5.80 ± 0.59 μg·mL^-1^ (95% CI: 5.02–6.58) in Δ*Nrf2*, representing a 3.8-fold increase in resistance (**Table 5**). The complemented strain regained sensitivity, with an EC_50_ of 1.42 ± 0.11 μg·mL^-1^ (95% CI: 1.21–1.63), not significantly different from WT.

Under normal conditions (without L-theanine), the Δ*Nrf2* mutant exhibited a slight reduction in mycelial growth rate (7.2% decrease at 7 days) and a modest decrease in toxin production (TEN: 12.3%, ALT: 8.7%, TeA: 10.1%) compared with WT, but these differences were not statistically significant (*p* > 0.05; **Supplementary Table S8**; **Supplementary Figure S7**), indicating that *Nrf2* deletion does not markedly affect basal growth or toxin biosynthesis in the absence of the compound.

### 3.5 *Nrf2* is required for toxin suppression and oxidative stress responses

Quantitative LC–MS/MS analysis revealed that L-theanine treatment at 2 μg·mL^-1^ significantly decreased the accumulation of three principal toxins, with amounts normalized to mycelial dry weight (**Table 10**; **Supplementary Figure S4**). Among these toxins, alternariol (ALT) exhibited the greatest inhibition rate (98.51%), followed by tentoxin (TEN) (69.39%) and tenuazonic acid (TeA) (48.99%). Consistent with these observations, RT-qPCR analysis of genes involved in toxin biosynthesis (at 12 h post-treatment) demonstrated marked downregulation: *PksA* (89.02%), *TES* (46.49%), and *PksJ* (40.13%). In the Δ*Nrf2* mutant, however, the inhibitory effect of L-theanine on toxin synthesis was almost entirely abolished: the inhibition rates for TEN, ALT, and TeA decreased to 12.64%, 0.49%, and 5.02%, respectively, and these decreases were accompanied by a reduction in the downregulation of related genes (*TES* 6.57%, *PksA* 2.12%, *PksJ* 7.68%) (**Table 10**).

**Table 10:** Inhibition of toxins by L-Theanine in different strains^a^.

Toxin	Strain	Content inhibition (%) ^b^	Gene ^c^	Gene downregulation (%) ^b^
TEN	WT	69.39 ± 0.80 a	*TES*	46.49 ± 0.50 a
TEN	Δ*Nrf2*	12.64 ± 0.21 b	*TES*	6.57 ± 0.18 b
TEN	Δ*Nrf2*-C	65.26 ± 0.70 a	*TES*	43.22 ± 0.53 a
ALT	WT	98.51 ± 0.92 a ^d^	*PksA*	89.02 ± 0.70 a
ALT	Δ*Nrf2*	0.49 ± 0.13 b	*PksA*	2.12 ± 0.31 b
ALT	Δ*Nrf2*-C	94.76 ± 0.90 a ^d^	*PksA*	90.31 ± 0.82 a
TeA	WT	48.99 ± 0.29 a	*PksJ*	40.13 ± 0.51 a
TeA	Δ*Nrf2*	5.02 ± 0.11 b	*PksJ*	7.68 ± 0.34 b
TeA	Δ*Nrf2*-C	44.96 ± 0.52 a	*PksJ*	40.29 ± 0.60 a

^a^L-theanine concentration 2 μg·mL^-1^ for 10 days (toxin accumulation) or 12 h (gene expression). Toxin amounts were normalized to mycelial dry weight. Values are mean ± SD (n = 4). ^b^ Inhibition and downregulation rates relative to those of the untreated control of the same strain. Different letters within the same toxin indicate significant differences among strains (*p* < 0.05, one-way ANOVA with Tukey’s test). ^c^ Gene names italicized. ^d^ For ALT, the inhibition rate in the complemented strain *ΔNrf2-C* (94.76%) was slightly lower than that in WT (98.51%); however, the two values were not statistically different (same letter, *p* > 0.05), and both were markedly higher than that in *ΔNrf2* (0.49%). This slight difference may reflect residual variation in the complemented strain and does not affect the conclusion that *Nrf2* is required for L-theanine-mediated ALT suppression.

As compared with the control group, *A. alternata* treated with L-theanine at 2 μg·mL^-1^ exhibited a pronounced rise in the level of intracellular reactive oxygen species (ROS), quantified as a 4.21-fold increase in relative fluorescence units (RFU) (**Supplementary Figure S3**; **Table 3**). Concurrently, the malondialdehyde (MDA) content markedly increased from 2.5 ± 0.3 to 12.1 ± 1.1 nmol·g^-1^ protein (~3.8-fold), indicating substantial membrane lipid peroxidation (*p* < 0.05). In parallel, the activities of key antioxidant enzymes were notably suppressed: superoxide dismutase (SOD) activity decreased from 50.2 ± 4.1 to 42.3 ± 3.9 U·mg^-1^ protein (15.7% reduction), whereas catalase (CAT) activity decreased more markedly from 15.6 ± 1.8 to 8.7 ± 1.2 U·mg^-1^ protein, amounting to a 44.2% reduction (*p* < 0.05) (**Table 3**). These findings suggest that L-theanine may exert its antifungal effect, at least in part, through inhibiting CAT activity, thereby impairing the detoxification of hydrogen peroxide (H_2_O_2_), which leads to the excessive accumulation of ROS and oxidative damage. A temporal analysis of glutathione (GSH) and glutathione S-transferase (GST) activities (**Supplementary Figure S5**; **Table 11**) revealed that during the initial phase (0–24 h), the GSH concentration decreased from 10.2 to 5.2 μmol·g^-1^ (**Table 11**, WT at 24 h), whereas the GST activity increased from 15.2 to 45.3 U·mg^-1^ (**Table 11**, WT at 24 h). This pattern suggests the induction of the glutathione-dependent detoxification mechanism. In the subsequent phase (24–48 h), the GSH concentration decreased to 3.1 μmol·g^-1^ (**Table 11**, WT at 48 h), accompanied by a reduction in the activity of GST to 9.6 U·mg^-1^ (**Table 11**, WT at 48 h), indicating the failure of the antioxidant defense system.

**Table 11:** Summary of GSH content and GST activity in different strains treated with L-Theanine.

Strain	Time	GSH (Control)^a^	GSH (Treated)^a^	GST (Control)^a^	GST (Treated)^a^
WT	0 h	10.2 ± 0.3 a	10.2 ± 0.3 a	15.2 ± 1.1 a	15.2 ± 1.1 b
WT	24 h	10.1 ± 0.3 a	5.2 ± 0.3 b	15.5 ± 1.0 a	45.3 ± 3.5 a
WT	48 h	10.0 ± 0.2 a	3.1 ± 0.4 c	14.9 ± 0.9 a	9.6 ± 1.5 c
Δ*Nrf2*	0 h	10.2 ± 0.3 a	10.2 ± 0.3 a	15.2 ± 1.1 a	15.2 ± 1.1 a
Δ*Nrf2*	24 h	10.2 ± 0.2 a	10.0 ± 0.5 a	15.8 ± 0.6 a	16.4 ± 2.0 a
Δ*Nrf2*	48 h	10.1 ± 0.3 a	9.7 ± 0.6 a	14.9 ± 0.9 a	15.7 ± 1.4 a
Δ*Nrf2*-C	0 h	10.2 ± 0.3 a	10.2 ± 0.3 a	15.2 ± 1.0 a	15.0 ± 1.2 b
Δ*Nrf2*-C	24 h	10.2 ± 0.2 a	5.2 ± 0.3 b	16.6 ± 0.8 a	48.3 ± 3.9 a
Δ*Nrf2*-C	48 h	10.1 ± 0.2 a	3.3 ± 0.2 c	14.7 ± 1.0 a	9.9 ± 1.2 c

^a^L-theanine concentration 2 μg·mL^-^¹. Values are mean ± SD (n = 3 independent biological replicates, each with three technical replicates). GSH units: μmol·g^-^¹; GST units: U·mg^-^¹. Different lowercase letters in the same column indicate significant differences among time points or between the control and treated at the same time (*p* < 0.05, two-way ANOVA/Tukey).

As shown in **Table 3**, L-theanine did not elicit a ROS burst in the Δ*Nrf2* mutant (RFU increase of only 1.14-fold); the MDA concentration slightly increased from 2.4 to 3.8 nmol·g^-1^, whereas the SOD and CAT activities remained stable (**Table 3**). Conversely, the response of the Δ*Nrf2*-C strain was comparable to that of the wild type (WT), characterized by a 4.14-fold increase in ROS (RFU), a substantial increase in MDA from 2.2 to 11.7 nmol·g^-1^, and significant reductions in SOD (52.6 to 45.3 U·mg^-1^) and CAT (16.0 to 9.1 U·mg^-1^) activities. These findings substantiate the critical role of Nrf2 in mediating L-theanine-induced oxidative stress. Time-course analyses of GSH and GST activity (**Supplementary Figure S5**; summarized in **Table 11**) confirmed that Δ*Nrf2* maintained stable GSH levels without GST induction, whereas the WT strain exhibited GSH depletion and subsequent GST activation followed by degradation.

Moreover, the expression of the antioxidant genes *CAT*, *SOD*, and *GPX* was quickly and transiently elevated in the WT, with increases of 8.2-, 10.5-, and 5.5-fold, respectively, at 3 h (**Table 4**; **Supplementary Figure S6**). However, this transcriptional upregulation was followed by a progressive decline in enzyme activities and subsequent oxidative damage. In the Δ*Nrf2* mutant, these genes showed a significantly weaker induction (1.8-, 2.5-, and 1.5-fold, respectively) compared with WT, suggesting that Nrf2 is required for the full transcriptional activation of these genes (**Table 4**; **Supplementary Figure S6**). Collectively, these findings demonstrate that Nrf2 is essential for the transcriptional activation of antioxidant defenses, but that L-theanine ultimately suppresses these enzyme activities, leading to oxidative damage.

## 4 Discussion

This study demonstrates that L-theanine, a metabolite detected in *Bacillus tequilensis* crude extract, exerts potent antifungal activity against *Alternaria alternata* through an Nrf2-dependent oxidative-stress response, accompanied by downregulation of ABC transporter and toxin biosynthesis genes. The deletion of *Nrf2* markedly reduced fungal sensitivity to L-theanine, abolished toxin suppression, and prevented oxidative stress responses, supporting the identification of Nrf2 as a candidate central node.

The observed EC_50_ values (1.54 μg·mL^-1^ for mycelial growth, 0.61 μg·mL^-1^ for spore germination) are particularly low, indicating that L-theanine is a highly effective antifungal metabolite. This potency is comparable to or exceeds that of many synthetic fungicides used in postharvest management (Wisniewski et al., 2016). Previous studies have reported antifungal activity of L-theanine against tea pathogens *Pestalotiopsis theae* (Zhang et al., 2024) and its role in enhancing plant resistance (Zhang et al., 2024). While those studies focused on metabolic pathway interference, our work reveals a distinct and complementary mechanism involving an Nrf2-dependent oxidative-stress response and the associated downregulation of transporter and toxin biosynthesis genes. This extends the understanding of L-theanine’s antifungal action by providing a detailed molecular mechanism in a major postharvest pathogen and by demonstrating significant reduction in mycotoxin production, which is critical for food safety. Because L-theanine was injected into the same wound that received the pathogen, and the solution remained within the wound without visible overflow, the reduced lesion expansion observed on detached fruit is consistent with the direct antifungal activity demonstrated *in vitro*.

The transcriptomic and WGCNA analyses revealed that L-theanine treatment leads to time-dependent downregulation of multiple ABC transporter genes, with Nrf2 as a key regulator. ABC transporters, especially those of the PDR and MRP families, are well known for mediating multidrug resistance and toxin efflux in fungi (Khunweeraphong and Kuchler, 2021; Paul and Moye-Rowley, 2014). Downregulation of these transporters would increase intracellular accumulation of L-theanine and other toxic metabolites, enhancing the antifungal effect. The strong co-expression between Nrf2 and ABC genes (r = 0.91) and the decreased Nrf2 expression upon treatment are consistent with a coordinated response, but they do not demonstrate that Nrf2 regulates ABC transporter genes. Whether Nrf2 mediates L-theanine-induced ABC transporter repression was not tested in this study. Whether L-theanine directly binds to Nrf2 or acts through upstream signalling pathways remains to be determined. This is consistent with the regulation of ABC genes by Yap1 in yeast (Wemmie et al., 1994; Miyahara et al., 1996). Notably, *AaPDR1* expression was unaffected in the Δ*Nrf2* mutant, despite being downregulated by L-theanine in WT. This observation suggests that additional transcription factors—such as AaCrz1 or the Zn_2_Cys_6_ factor AaZ6C1—may also contribute to its regulation. We hypothesize that *AaPDR1* may be regulated through calcium signalling pathways or Zn_2_Cys_6_ transcription factors, whereas Nrf2 may be involved in the regulation of PDR5 homologues and MRP family transporters. This indicates a complex regulatory network, possibly integrating calcium signalling and MAPK pathways (Franci et al., 2024).

It is important to address an apparent paradox in our data. The Δ*Nrf2* mutant showed reduced basal expression of several ABC transporter genes, yet it was more resistant to L-theanine than the WT. This indicates that ABC transporter expression alone does not determine L-theanine sensitivity. Instead, the resistance of Δ*Nrf2* is primarily attributable to the loss of the Nrf2-dependent oxidative-stress response: the mutant failed to mount the transient antioxidant transcriptional activation and did not exhibit the subsequent ROS burst, lipid peroxidation, or antioxidant enzyme suppression. In this framework, ABC transporter repression was not tested for Nrf2 dependence and is therefore treated as a correlative observation, not as a demonstrated downstream output of Nrf2. We do not claim direct transcriptional regulation of ABC transporters by Nrf2, and we have not tested whether Nrf2 is required for L-theanine-induced repression of these genes. Whether Nrf2 directly binds to ABC transporter promoters remains to be tested by ChIP-seq or EMSA.

The near-complete loss of toxin suppression in Δ*Nrf2* underscores the essential role of Nrf2 in mediating L-theanine’s effect on secondary metabolism. Toxin production in *Alternaria* is known to be regulated by oxidative stress and transcription factors (Montibus et al., 2015). Our data show that L-theanine reduces the expression of toxin biosynthetic genes (*PksA*, *TES*, *PksJ*) in an Nrf2-dependent manner, linking oxidative stress signalling to secondary metabolite repression. The high inhibition of alternariol (98.51%) is particularly promising for food safety, as alternariol is a potent genotoxin (Prencipe et al., 2023). Since L-theanine strongly inhibits fungal growth, we normalized toxin amounts to mycelial dry weight, confirming that the reduction is not solely due to growth inhibition.

The oxidative stress response induced by L-theanine is also Nrf2-dependent. In WT, we observed a significant ROS burst, lipid peroxidation (increased MDA), and suppression of CAT, SOD, and GPX enzyme activities. Importantly, the WT showed a transient upregulation of antioxidant genes at 3 h (8.2-, 10.5-, and 5.5-fold for *CAT*, *SOD*, and *GPX*, respectively), representing an attempted cellular defense. However, this transcriptional activation was followed by a progressive decline in enzyme activities and subsequent oxidative damage, indicating that the sustained suppression of Nrf2 eventually overwhelms the antioxidant system. In Δ*Nrf2*, both the transcriptional activation and the subsequent enzyme suppression were absent, confirming that Nrf2 is required for the initial transcriptional response. The antifungal effect of L-theanine reflects the integrated consequence of the Nrf2-dependent transcriptional program. ABC transporter repression was not tested for Nrf2 dependence and is treated as a correlative observation in this study. The biphasic GSH/GST response—initial GST induction followed by GSH depletion and GST decline—indicates that the antioxidant defense is overwhelmed after 24 h in WT, whereas Δ*Nrf2* maintains stable GSH levels without GST induction.

The identification of the fungal transcription factor as “Nrf2” warrants justification. Based on sequence homology and domain architecture, the gene we designated *Nrf2* (locus tag: AaNrf2_001; **Supplementary Table S9**) encodes a bZIP transcription factor that shares 58% similarity with Yap1 from *S. cerevisiae* and contains a conserved N-terminal nuclear localization signal (KRPRKLELEDELKRRR). This gene is a homologue of yeast Yap1/Nrf2-like oxidative-stress regulators; we have included the locus identifier and sequence characteristics in the **Supplementary Materials**. To avoid confusion with mammalian Nrf2, we note that AaNrf2_001 is a fungal Yap1-like transcription factor. The name “Nrf2” is used here operationally because of its homology to the Yap1/Nrf2 family; however, it should not be interpreted as a direct orthologue of mammalian Nrf2. Future studies using promoter-binding assays (e.g., ChIP-seq) would be needed to confirm direct transcriptional regulation of ABC transporters by Nrf2.

Several limitations should be acknowledged. First, we have not yet determined whether L-theanine directly binds to Nrf2 or acts through upstream signalling (e.g., MAPK pathways) (Franci et al., 2024; Sun et al., 2009). Future studies using surface plasmon resonance, molecular docking, and phosphoproteomics will clarify this. Second, our experiments were conducted under controlled laboratory conditions and in detached fruit; field trials and storage-house trials are needed to evaluate the efficacy and safety of L-theanine in practical pear storage. Third, the regulatory network may involve additional transcription factors and post-translational modifications that fine-tune the response. Fourth, the potential for cross-resistance and long-term stability of L-theanine in the environment should be investigated. Fifth, practical application would require assessment of stability, persistence, phytotoxicity, fruit quality, effective concentration under storage conditions, residue and safety evaluation, and scale-up production. Sixth, the extraction procedure for the crude metabolite extract was not fully documented for the methanol-based recovery step; future work should use a fully validated extraction protocol if the crude extract is to be developed further. This does not affect the reported antifungal concentrations or mechanistic conclusions, because all antifungal and mechanistic assays used commercial L-theanine standard. Seventh, we have not tested whether Nrf2 mediates L-theanine-induced repression of ABC transporter genes; this requires a full factorial experiment. Eighth, because no internal standard was used and no full validation (LOD, LOQ, recovery, precision) was performed, the LC-MS/MS data confirm the presence of L-theanine in the crude extract and provide the concentration used for dosing, but do not constitute a fully validated quantitative method. Therefore, we describe L-theanine as a promising antifungal candidate for further development, pending further evaluation.

In conclusion, we have elucidated a mechanism by which L-theanine detected in *B. tequilensis* inhibits *A. alternata*: an Nrf2-dependent oxidative-stress response, accompanied by downregulation of ABC transporter and toxin biosynthesis genes. This positions Nrf2 as a promising target for antifungal development and L-theanine as a sustainable candidate for further investigation in postharvest disease management. Our findings also provide a foundation for exploring similar mechanisms in other fungal pathogens and for engineering biocontrol strains with enhanced metabolite production.

## 5 Conclusion

This study demonstrates that L-theanine, a metabolite detected in *Bacillus tequilensis* crude extract, exhibits potent antifungal activity against *Alternaria alternata* through an Nrf2-dependent oxidative-stress response, accompanied by downregulation of ABC transporter and toxin biosynthesis genes. Nrf2-dependence of the ABC transporter repression was not tested in this study. The identification of Nrf2 as a central regulatory hub provides a molecular basis for understanding the antifungal action of L-theanine and establishes it as a promising candidate for further development in the integrated management of postharvest pear diseases. Validation under laboratory and detached-fruit conditions supports the need for further storage, safety, stability, phytotoxicity, and fruit-quality studies before practical application.

## Data Availability

The raw RNA seq data generated in this study have been deposited in the National Center for Biotechnology Information (NCBI) Sequence Read Archive (SRA) under BioProject accession number PRJNA895027. All other data are contained within the article and **Supplementary Material**.
