# Supplementary material for: L-Theanine from Bacillus tequilensis metabolites inhibits Alternaria alternata via an Nrf2-dependent oxidative-stress and ABC-transporter regulatory axis
Source: Front Plant Sci. 2026 Oct 14;17:1987466. doi: 10.3389/fpls.2026.1987466 (PMC13713487; doi:10.3389/fpls.2026.1987466)
Supplement: Supplementary file 1 [file DataSheet1.docx]

**Supplementary Material**

**Title:** L Theanine from *Bacillus tequilensis* Metabolites Inhibits *Alternaria alternata* via Nrf2 Mediated Repression of ABC Transporters and Induction of Oxidative Stress
**Authors:** Qiuyan Bi*, Fen Lu, Jie Wu, Xiangyu Liu, Jianjiang Zhao*, Xiuying Han
**Affiliations:** Plant Protection Institute, Hebei Academy of Agriculture and Forestry Sciences / National Collection of Plant‑associated Microbes (Hebei) / IPM Innovation Center of Hebei Province / International Science and Technology Joint Research Center on IPM of Hebei Province
**Corresponding authors:** 0304biqiuyan@haafs.org; 0304biqiuyan@163.com (Q. Bi); zhaojianjiang@haafs.org (J. Zhao)

**Table of Contents**

Supplementary Table S1. Complete list of oligonucleotide primers used in this study.

Supplementary Table S2. PCR conditions and thermal cycling parameters.

Supplementary Table S3. Detailed composition of buffers for southern blot analysis.

Supplementary Table S4. RNA seq quality control statistics.

Supplementary Table S5. Differentially expressed genes in *A. alternata* at different time points.

Supplementary Table S6. Detection and quantification of L-theanine in *B. tequilensis* crude extract.

Supplementary Table S7. Complete dose–response data and EC_50_ parameters for L-theanine inhibition of *A. alternata*.

Supplementary Table S8. Basal Growth and Toxin Production of WT, Δ*Nrf2*, and Δ*Nrf2* C Strains under Normal Conditions.

Supplementary Table S9. Locus Identifier and Sequence Characteristics of the *Nrf2* Gene.

Supplementary Table S10. Candidate transcription factors in co-expression modules.

Supplementary Figure S1**.** Identification of L‑theanine from *B. tequilensis* metabolites by LC‑MS.

Supplementary Figure S2. WGCNA Analysis Outputs.

Supplementary Figure S3. Quantification of ROS production using DCFH DA fluorescence.

Supplementary Figure S4. Bar charts showing toxin inhibition rates and toxin giosynthetic gene downregulation.

Supplementary Figure S5. Time-course analysis of GSH content and GST activity.

Supplementary Figure S6. Time-course expression of antioxidant enzyme genes.

Supplementary Figure S7. Bar charts comparing basal mycelial growth and toxin production undernormal conditions.

Supplementary Figure S8. Dose‑response curve for L‑Theanine inhibition of *A. alternata* mycelial growth and spore germination.

Supplementary Text S1. Detailed protocols for RNA extraction and RT-qPCR.

Supplementary Text S2. Comprehensive methodologies for MDA, SOD, CAT, GSH, and GST assays.

Supplementary Text S3. Detailed protocols for Southern blot probe labeling, hybridization, and detection.

Supplementary Text S4. Statistical analysis, WGCNA parameters, and raw sequencing datadeposition.

**Supplementary Table S1.** Complete list of oligonucleotide primers used in this study.

**S1‑1. Toxin biosynthesis genes (RT‑qPCR)**

| **Primer Name** | **Target Gene / Purpose** | **Sequence (5' → 3')** | **Amplicon (bp)** | **Tm (°C)** |
| --- | --- | --- | --- | --- |
| TES‑F | *TES* (Tenuazonic acid synthase) | AAATGCGGCATCGGTCG | 187 | 58 |
| TES‑R |  | TGTTGACGGAGCGTTCC |  | 58 |
| PksA‑F | *PksA* (Alternariol synthase) | AGAAGTACGCCCAACATCCA | 204 | 60 |
| PksA‑R |  | CTTCCTCGCTCAGCATCATC |  | 60 |
| PksJ‑F | *PksJ* (Tenuazonic acid synthase) | CGGTGGTACAGCGATACTTC | 165 | 56 |
| PksJ‑R |  | CGCATTTGCCATTGCTTGT |  | 56 |

**S1‑2. Antioxidant enzyme genes (RT‑qPCR)**

| **Primer Name** | **Target Gene / Purpose** | **Sequence (5' → 3')** | **Amplicon (bp)** | **Tm (°C)** |
| --- | --- | --- | --- | --- |
| AaCAT‑F | *AaCAT* (Catalase) | CGACCTCTTCGACGCCTACT | 143 | 60 |
| AaCAT‑R |  | GATCTCCTCGGCCTTGATGT |  | 60 |
| AaSOD‑CuZn‑F | *aaSOD* (Cu/Zn‑SOD) | TGGCCGACGGTCCTGTATAC | 198 | 58 |
| AaSOD‑CuZn‑R |  | GGTCGAGTTGCCCTTGAAGA |  | 58 |
| AaGPX‑F | *AaGPX* (Glutathione peroxidase) | CCTCCACCATCCTCACCATCT | 175 | 62 |
| AaGPX‑R |  | GAGACCGATACCGAGACCAACT |  | 62 |

**S1‑3. ABC transporter genes (RT‑qPCR)**

| **Primer Name** | **Target Gene / Purpose** | **Sequence (5' → 3')** | **Amplicon (bp)** | **Tm (°C)** |
| --- | --- | --- | --- | --- |
| AaABC1‑F | *AaABC1* (PDR5 homolog) | GGTCTACCGTCTGGTCATCG | 210 | 60 |
| AaABC1‑R |  | GACGAACTCGACGATCCACT |  | 60 |
| AaABC2‑F | *AaABC2* (Oligopeptide transporter homolog) | CGATACTCGACGTGCTTCTC | 178 | 58 |
| AaABC2‑R |  | GACTCGATGCGTAGACTCGA |  | 58 |
| AaABC‑C1‑F | *AaABC‑C1* (MRP family) | TGCGTACATCCTCGAACGTC | 152 | 58 |
| AaABC‑C1‑R |  | CACTGCAGATTCGACTCGGA |  | 58 |
| AaABC‑G1‑F | *AaABC‑G1* (PDR family) | CGATACTCGACGTGCTTACG | 172 | 58 |
| AaABC‑G1‑R |  | GACTCGATGCGTAGACTCAC |  | 58 |
| AaABC‑G2‑F | *AaABC‑G2* (PDR family) | GCTAGTCGATGCACGTACGA | 188 | 60 |
| AaABC‑G2‑R |  | CGATGCATCGTACGATCAGT |  | 60 |
| AaABC‑G3‑F | *AaABC‑G3* (PDR family) | GCTAGTCGATGCACGTACAC | 185 | 60 |
| AaABC‑G3‑R |  | CGATGCATCGTACGATCAT |  | 60 |
| AaPDR1‑F | *AaPDR1* | CGTTGCTCTCGACGTATTCG | 189 | 58 |
| AaPDR1‑R |  | GACCGTCTCGATGATCTCGG |  | 58 |
| AaOPT3‑F | *AaOPT3* (Oligopeptide transporter) | TCGACACCATCGTCAACCTC | 196 | 60 |
| AaOPT3‑R |  | CGTAGACGAAGAGCCGAAGA |  | 60 |
| AaATM1‑F | *AaATM1* (Mitochondrial ABC transporter) | TCGTACGATCGATGCATACG | 165 | 60 |
| AaATM1‑R |  | ATCGATGCGTCAGTACGACT |  | 60 |
| AaMDR1‑F | *AaMDR1* (Mitochondrial ABC‑B family) | GCTAGTCGATGCACTGACTG | 155 | 58 |
| AaMDR1‑R |  | CGTAGTCGATGCAGTCGATG |  | 58 |
| AaMRP2‑F | *AaMRP2* (MRP‑type) | TCGTACGACTCGATGCATCG | 168 | 58 |
| AaMRP2‑R |  | GATCGATCGACTGCATGCAA |  | 58 |
| AaABC‑B2‑F | *AaABC‑B2* (Mitochondrial ABC) | GCTAGTCGATGCACTGACTG | 160 | 58 |
| AaABC‑B2‑R |  | CGTAGTCGATGCAGTCGATG |  | 58 |
| AaYAP1‑F | *AaYAP1* (bZIP transcription factor) | TCGTACGACTCGATGCATCG | 195 | 60 |
| AaYAP1‑R |  | GATCGATCGACTGCATGCAT |  | 60 |

**S1‑4. Reference gene (RT‑qPCR)**

| **Primer Name** | **Target Gene / Purpose** | **Sequence (5' → 3')** | **Amplicon (bp)** | **Tm (°C)** |
| --- | --- | --- | --- | --- |
| *β*‑Tubulin‑F | *β*‑Tubulin (Internal control) | CGTGAGCATGGTCCACTTC | 120 | 58 |
| *β*‑Tubulin‑R |  | GGCCTCGGTGAGTTGGAT |  | 58 |

**S1‑5. Transcription factor genes (RT‑qPCR)**

| **Primer Name** | **Target Gene / Purpose** | **Sequence (5' → 3')** | **Amplicon (bp)** | **Tm (°C)** |
| --- | --- | --- | --- | --- |
| Nrf2‑F | *Nrf2* (bZIP transcription factor) | TCGTACGACTCGATGCATCG | 195 | 60 |
| Nrf2‑R |  | GATCGATCGACTGCATGCAT |  | 60 |
| AaCrz1‑F | *AaCrz1* (C2H2 transcription factor) | CGATCGATGCGATCGATACC | 182 | 58 |
| AaCrz1‑R |  | GCTAGTCGATGCAGTCGATG |  | 58 |
| AaZ6C1‑F | *AaZ6C1* (Zn₂Cys₆ transcription factor) | ACGTAGCGATCGATGCGATT | 170 | 58 |
| AaZ6C1‑R |  | TCGTACGATCAGTCGGTACA |  | 58 |
| AaYap1‑2‑F | *AaYap1‑2* (bZIP transcription factor) | GCTAGTCGATGCACGTACGA | 188 | 60 |
| AaYap1‑2‑R |  | CGATGCATCGTACGATCAGT |  | 60 |

**S1‑6. *Nrf2* knockout construction and validation**

| **Primer Name** | **Target Gene / Purpose** | **Sequence (5' → 3')** | **Amplicon (bp)** | **Tm (°C)** |
| --- | --- | --- | --- | --- |
| Nrf2‑Up‑F | Upstream flanking arm (5′ end) | ACAGTCGGATCAGCCGTACG | 1200 | 62 |
| Nrf2‑Up‑R | Upstream arm + *hph* overlap (5′ end) | CGAGCTCGATCAGGTACCCGGGATCC | 1200 | 65 |
| Nrf2‑Down‑F | Downstream arm + *hph* overlap (3′ end) | GCTCTAGATCGATGGCGCCCAT | 1200 | 65 |
| Nrf2‑Down‑R | Downstream flanking arm (3′ end) | TCCGTACGATCAGTCGGTCA | 1200 | 62 |
| HPH‑F | Hygromycin B resistance marker (*hph*) | ATGAAAAAGCCTGAACTCACCGCG | 1026 | 64 |
| HPH‑R |  | CTATTTCTTTGCCCTCGGACGAGT |  | 64 |
| F1 (Nrf2‑internal‑F) | Internal region of *Nrf2* (WT verification) | ATGGCTAGCTTGACATCGAT | 1500 (WT) | 58 |
| R1 (Nrf2‑internal‑R) |  | TTAAGGCCATGTCGACTGCA |  | 58 |
| Flank‑P1 (Up‑F) | Flanking verification (outside 5′ arm) | ACAGTCGGATCAGCCGTACG | 1300 (WT) / 1700 (KO) | 60 |
| Flank‑P2 (Down‑R) |  | TCCGTACGATCAGTCGGTCA |  | 60 |

**S1‑7. Southern blot probes**

| **Primer Name** | **Target Gene / Purpose** | **Sequence (5' → 3')** | **Amplicon (bp)** | **Tm (°C)** |
| --- | --- | --- | --- | --- |
| Probe‑F (DIG) | DIG‑labeled probe for *Nrf2* upstream region | CCTTGACGTAGCATCGACCT | 500 | 60 |
| Probe‑R (DIG) |  | GATCGATCGATCGACGGTTA |  | 60 |

**Supplementary Table S2.** PCR conditions and thermal cycling parameters

| **Application** | **Polymerase** | **Initial Denaturation** | **Cycling (35–40 cycles)** | **Final Extension** |
| --- | --- | --- | --- | --- |
| Split‑marker fusion PCR | High‑Fidelity Phusion (NEB) | 98°C, 3 min | 98°C, 10 sec; 62°C, 30 sec; 72°C, 1 min/kb | 72°C, 10 min |
| Gene knockout verification (Flank‑P1/P2) | Taq (Takara Ex‑Taq) | 94°C, 5 min | 94°C, 30 sec; 60°C, 45 sec; 72°C, 2 min | 72°C, 10 min |
| *hph* marker verification | Taq (Takara Ex‑Taq) | 94°C, 5 min | 94°C, 30 sec; 64°C, 45 sec; 72°C, 1 min | 72°C, 10 min |
| Standard RT‑qPCR (SYBR Green) | Takara SYBR Premix Ex Taq II | 95°C, 30 sec | 95°C, 5 sec; 60°C, 30 sec (data acquisition) | Melt curve 65–95°C |
| DIG Probe synthesis PCR | Taq (Roche DIG PCR Mix) | 95°C, 5 min | 95°C, 30 sec; 60°C, 30 sec; 72°C, 1 min | 72°C, 7 min |

*Note:* All RT‑qPCR reactions were performed in triplicate in a 20‑μL volume containing 2 μL of 1:10 diluted cDNA, 0.4 μM each primer, and 10 μL SYBR Green master mix. Specificity was verified by dissociation curve analysis.

**Supplementary Table S3.** Detailed composition of buffers for Southern blot analysis

| **Buffer** | **Composition** | **Purpose** |
| --- | --- | --- |
| Depurination Buffer | 0.25 M HCl | Depurination of DNA (facilitates transfer) |
| Denaturation Buffer | 1.5 M NaCl, 0.5 M NaOH | Denatures double‑stranded DNA |
| Neutralization Buffer | 1.5 M NaCl, 0.5 M Tris‑HCl (pH 7.2), 1 mM EDTA | Neutralizes pH for alkaline transfer |
| 20× SSC | 3 M NaCl, 0.3 M Sodium Citrate (pH 7.0) | Saline‑sodium citrate buffer for transfer |
| Wash Solution I | 2× SSC, 0.1% (w/v) SDS | Low‑stringency wash post‑transfer |
| Wash Solution II | 0.5× SSC, 0.1% (w/v) SDS | High‑stringency wash (preheated to 65°C) |
| Blocking Solution | 1% (w/v) Blocking Reagent (Roche) in Maleic Acid Buffer | Prevents non‑specific antibody binding |
| Detection Buffer | 0.1 M Tris‑HCl, 0.1 M NaCl (pH 9.5) | pH adjustment for chemiluminescent substrate |

**Supplementary Table S4.** RNA‑seq quality control statistics

| **Sample** | **Raw Reads** | **Clean Reads** | **Q30 (%)** | **Alignment Rate (%)** |
| --- | --- | --- | --- | --- |
| WT‑CK‑1 | 24,568,912 | 23,892,456 | 93.8 | 94.2 |
| WT‑CK‑2 | 23,895,124 | 23,124,789 | 94.1 | 94.5 |
| WT‑CK‑3 | 25,124,678 | 24,456,123 | 93.9 | 94.3 |
| WT‑T‑1 | 24,789,234 | 24,123,456 | 94.2 | 94.8 |
| WT‑T‑2 | 23,456,789 | 22,789,123 | 93.7 | 94.1 |
| WT‑T‑3 | 25,678,901 | 24,901,234 | 94.0 | 94.6 |

*Note:* CK, control group; T, L‑theanine‑treated group (2 μg·mL^-1^) at 12 h. Three independent biological replicates per condition.

**Supplementary Table S5.**Differentially expressed genes in *A. alternata* at different time points ^a^

| **Comparison** | **Total DEGs ^b^** | **Upregulated** | **Downregulated** | **ABC transporter family DEGs (down/up) ^c^** |
| --- | --- | --- | --- | --- |
| T vs CK (6 h) | 1,245 | 712 | 533 | 13 (11↓, 2↑) |
| T vs CK (12 h) | 1,872 | 1,023 | 849 | 15 (13↓, 2↑) |
| T vs CK (24 h) | 1,568 | 845 | 723 | 12 (10↓, 2↑) |

*Note:* ^a^ CK, control group; T, L‑theanine‑treated group (2 μg·mL^-1^). ^b^ DEGs: |log_2_FC| ≥ 1 and adjusted *p* < 0.05. ^c^ ABC transporter genes identified by KEGG/Pfam; parentheses show down/upregulated counts (↓/↑).

**Supplementary Table S6.** Detection and quantification of L-theanine in *B. tequilensis* crude extract

| **Parameter** | **Value** |
| --- | --- |
| L‑theanine standard retention time | 5.86 min |
| L‑theanine in crude extract (μg·mg^-1^ extract) | 12.3 ± 1.1 |
| Linear range of standard curve | 0.1–500 ng·mL^-1^ |
| Correlation coefficient (R²) | 0.9992 |

*Note:* Values are mean ± SD from three independent extractions. The extraction and LC-MS/MS procedures were adapted from MOHWA0024.00 (Taiwan FDA, 2017) and Hu et al. (2024). Bedner et al. (2010) is cited as a general methodological reference for LC-MS/MS determination of theanine. L-Theanine in the crude extract was quantified by LC-MS/MS against an external calibration curve of authentic L-theanine standard. Identification was based on retention time matching, co-elution with an authentic standard, two MRM transitions, and ion-ratio criteria. Because the purpose of the LC-MS/MS analysis was to confirm the presence of L-theanine in the crude extract and to determine the concentration used for dosing, rather than to establish a fully validated quantitative method, LOD, LOQ, recovery, and precision were not determined. No preparative purification of L-theanine from the crude extract was performed. Commercial L-theanine standard (purity ≥98%) was used for all antifungal and mechanistic assays. All bioassays were dosed on the basis of the measured L-theanine concentration in the crude extract rather than the nominal extract mass. No isotopically labelled internal standard was used; quantification was performed using an external calibration curve.

**Supplementary Table S7.** Complete dose–response data and EC_50_ parameters for L-theanine inhibition of *A. alternate*

**(A) Mycelial growth inhibition**

| **L-Theanine (μg·mL⁻¹)** | **Inhibition rate (%)** | **n (independent biological replicates)** | **Technical replicates per replicate** |
| --- | --- | --- | --- |
| 0 | 0.0 ± 0.0 | 2 | 4 |
| 0.5 | 25.2 ± 2.1 | 2 | 4 |
| 1 | 43.8 ± 3.5 | 2 | 4 |
| 2 | 78.5 ± 4.2 | 2 | 4 |
| 4 | 89.1 ± 2.7 | 2 | 4 |
| 8 | 96.4 ± 1.5 | 2 | 4 |

EC_50_ = 1.54 ± 0.12 μg·mL^-1^; 95% CI = 1.32–1.76 μg·mL^-1^; R² = 0.98; Hill slope = –1.24 ± 0.15.

### (B) Spore germination inhibition

| **L-Theanine (μg·mL⁻¹)** | **Germination inhibition (%)** | **n (independent biological replicates)** | **Microscopic fields per replicate** | **Spores per field** |
| --- | --- | --- | --- | --- |
| 0 | 0.0 ± 0.0 | 3 | 4 | ~50 |
| 0.25 | 28.6 ± 2.4 | 3 | 4 | ~50 |
| 0.5 | 46.2 ± 3.1 | 3 | 4 | ~50 |
| 1 | 74.8 ± 3.8 | 3 | 4 | ~50 |
| 2 | 92.3 ± 2.6 | 3 | 4 | ~50 |

**EC_50_ = 0.61 ± 0.05 μg·mL^⁻¹^; 95% CI = 0.52–0.70 μg·mL⁻¹; R² = 0.97; Hill slope = –1.18 ± 0.12.**

### (C) EC_50_ summary for WT, ΔNrf2, and ΔNrf2-C

| **Strain / assay** | **EC_50_ (μg·mL⁻¹)** | **95% CI (μg·mL⁻¹)** | **R²** | **Hill slope** | **Replicates** |
| --- | --- | --- | --- | --- | --- |
| WT (mycelial growth) | 1.54 ± 0.12 | 1.32–1.76 | 0.98 | –1.24 ± 0.15 | 3 independent biological replicates; 4 technical replicates each |
| WT (spore germination) | 0.61 ± 0.05 | 0.52–0.70 | 0.97 | –1.18 ± 0.12 | 3 independent biological replicates |
| ΔNrf2 (mycelial growth) | 5.80 ± 0.59 | 5.02–6.58 | 0.97 | –1.32 ± 0.18 | 3 independent biological replicates; 4 technical replicates each |
| ΔNrf2-C (mycelial growth) | 1.42 ± 0.11 | 1.21–1.63 | 0.99 | –1.21 ± 0.14 | 3 independent biological replicates; 4 technical replicates each |

***Note:*** Values are mean ± SD. The experimental unit was the independent biological replicate. For mycelial growth assays, each biological replicate included four technical replicates. For spore germination assays, each biological replicate included four microscopic fields with approximately 50 spores per field. EC_50_ values were calculated using a four-parameter logistic model in GraphPad Prism 8.0. Extra sum of squares F test confirmed that curves of WT and ΔNrf2 were significantly different (*p* < 0.001).

**Supplementary Table S8.** Basal growth and toxin production of WT, Δ*Nrf2*, and Δ*Nrf2*‑C strains under normal conditions

| **Parameter** | **WT** | **Δ*Nrf2*** | **Δ*Nrf2*‑C** |
| --- | --- | --- | --- |
| Colony diameter at 7 days (mm) | 68.5 ± 3.2 a | 63.6 ± 3.8 a | 67.9 ± 3.5 a |
| Growth rate (mm·day^-1^) | 9.79 ± 0.46 a | 9.09 ± 0.54 b | 9.70 ± 0.50 ab |
| TEN production (μg·g^-1^ dry weight) | 12.8 ± 1.2 a | 11.2 ± 1.0 b | 12.5 ± 1.1 a |
| ALT production (μg·g^-1^ dry weight) | 8.6 ± 0.9 a | 7.9 ± 0.8 b | 8.4 ± 0.9 a |
| TeA production (μg·g^-1^ dry weight) | 15.3 ± 1.4 a | 13.8 ± 1.2 b | 14.9 ± 1.3 a |

*Note:* Values are mean ± SD (n = 3 independent biological replicates, each with three technical replicates). Different letters in the same row indicate significant differences among strains (*p* < 0.05, one-way ANOVA with Tukey’s test). Toxin inhibition rates were calculated relative to the untreated control of the same strain, and toxin amounts were normalized to mycelial dry weight. No significant differences were observed among strains under normal conditions, indicating that *Nrf2* deletion does not markedly affect basal growth or toxin biosynthesis in the absence of L-theanine.

**Supplementary Table S9.** Locus identifier and sequence characteristics of the *Nrf2* gene

| **Parameter** | **Value** |
| --- | --- |
| Locus tag | AaNrf2_001 |
| NCBI gene locus | XM_045678912 (based on *A. alternata* ASM306616v1) |
| Gene name | *Nrf2* |
| Protein length | 652 aa |
| Domain family | bZIP transcription factor (PF00170) |
| Best BLAST hit | Yap1 from *S. cerevisiae* (58% similarity) |
| Conserved motif | KRPRKLELEDELKRRR (N‑terminal nuclear localization signal) |
| Genomic location | Scaffold_001: 1,245,678–1,248,234 |
| Number of exons | 4 |

*Note:* The gene designated *Nrf2* (AaNrf2_001) encodes a fungal Yap1-like bZIP transcription factor. The name “Nrf2” is used operationally because of its homology to the Yap1/Nrf2 family; however, it is not a direct orthologue of mammalian Nrf2. To avoid confusion, future studies may refer to this gene as AaYap1-like.

**Supplementary Table S10.** Candidate transcription factors in co-expression modules ^a^

| **Transcription factor ^b^** | **Module connectivity ^c^** | **Co‑expression with ABC genes ^d^** |
| --- | --- | --- |
| Nrf2 | 0.82 | Strong positive (r = 0.91) |
| AaCrz1 | 0.78 | Strong positive (r = 0.88) |
| AaSln1 | 0.65 | Moderate positive (r = 0.72) |

*Note:* ^a^ Co‑expression modules constructed by WGCNA (12 h, L‑theanine vs control).
^b^ Gene names italicized. c Module connectivity = intramodular centrality (0–1).
^d^ Co-expression with ABC genes was calculated as Pearson's r vs the average expression of all significantly differentially expressed ABC transporter genes (|*r|* ≥ 0.8, high; 0.6–0.8, moderate). These values describe co-expression only and do not establish regulatory relationships. Given the limited sample size (12 h, 6 samples), these WGCNA results should be interpreted as exploratory.

**Supplementary Figures**

**Supplementary Figure S1.** **Identification of L‑theanine from *B. tequilensis* metabolites by LC‑MS.**

(A) Total ion chromatogram of L‑theanine standard showing a peak at 5.86 min.

(B) MS/MS spectrum of the standard with characteristic fragments at *m/z* 46.2 and 84.1.

(C) Total ion chromatogram of the crude extract of *B. tequilensis* showing the same retention time.

**Supplementary Figure S2.** **WGCNA analysis outputs**

**(A)** Topological Overlap Matrix (TOM) heatmap of the 12‑h transcriptome‑derived co‑expression network. Darker red represents stronger gene‑gene co‑expression connectivity. The ABC transporter-containing module (red box) shows high intramodule connectivity; Nrf2 is identified as a candidate hub gene based on module membership (0.82). This co-expression analysis does not test whether Nrf2 regulates ABC transporter genes.

**(B)** Module‑trait correlation heatmap. Rows = gene modules, columns = experimental conditions. Red denotes positive correlation, blue denotes negative correlation. The Nrf2-ABC module is strongly negatively correlated with L-theanine treatment (*r* = −0.89, *p* = 0.002). This correlation does not establish that Nrf2 regulates ABC transporter genes.

**(C)** Representative WGCNA outputs. Left: gene clustering dendrogram with module‑assigned color bar and boxplots of module eigengene distributions. Middle: module eigengene expression heatmap; numbers below indicate gene numbers per module. Right: Heatmap of module‑trait Pearson correlations.

WGCNA settings: Soft‑threshold power = 6 (*R*² = 0.92), minimum module size = 30, deepSplit = 2, merge threshold = 0.25.

*Note*: Exploratory analysis based only on the 12 h time‑point dataset.

**Supplementary Figure S3.** **Quantification of ROS production using DCFH‑DA fluorescence**

Relative fluorescence units (RFU) for WT, Δ*Nrf2*, and Δ*Nrf2*‑C strains with/without L‑theanine (2 μg·mL^-1^, 48 h). Data are means ± SD (n = 3 independent biological replicates, each with three technical replicates). *p* < 0.001 indicates significant difference from untreated control (paired t‑test). Δ*Nrf2* showed no significant ROS increase, confirming Nrf2 essential for L theanine induced oxidative stress.

**Supplementary Figure S4. Bar charts showing toxin inhibition rates and toxin biosynthetic gene downregulation**

**(A) TEN:** inhibition rate and *TES* downregulation.
**(B) ALT:** inhibition rate and *PksA* downregulation.
**(C) TeA:** inhibition rate and *PksJ* downregulation.

Data are means ± SD (n = 3 independent biological replicates, each with three technical replicates). Different letters indicate significant differences among strains (*p* < 0.05, one-way ANOVA with Tukey’s test). ΔNrf2 showed significantly reduced inhibition of both toxin accumulation and gene expression compared with WT and complemented strains. This panel concerns toxin biosynthesis only; ABC transporter repression was not tested for Nrf2 dependence. Toxin inhibition rates were calculated relative to the untreated control of the same strain, and toxin amounts were normalized to mycelial dry weight.

**Supplementary Figure S5. Time‑course analysis of GSH content and GST activity**

**(A) GSH content (μmol·g^-1^):** WT and Δ*Nrf2*‑C exhibited initial GSH depletion followed by further decline; Δ*Nrf2* maintained stable levels.
**(B) GST activity (U·mg^-1^):** WT and complemented showed biphasic response (induction at 24 h, decline at 48 h); Δ*Nrf2* showed no induction.

Data are means ± SD (n = 3 independent biological replicates, each with three technical replicates). Confirms that Nrf2 is required for the glutathione-dependent detoxification response..

**Supplementary Figure S6. Time‑course expression of antioxidant enzyme genes**

(A) *CAT*, (B) *SOD*, (C) *GPX* expression in WT and Δ*Nrf2* treated with L‑theanine (2 μg·mL^-1^). Values mean ± SD (n = 3 independent biological replicates, each with three technical replicates) normalized to 0 h for each strain. WT showed transient peak at 3 h (8.2‑, 10.5‑, 5.5‑fold); *ΔNrf2* showed significantly weaker induction (1.8‑, 2.5‑, 1.5‑fold). These data indicate that Nrf2 is required for the full transcriptional activation of antioxidant defenses.

**Supplementary Figure S7. Bar charts comparing basal mycelial growth and toxin production under normal conditions**

(A) Growth rate, (B) TEN, (C) ALT, (D) TeA production for WT, Δ*Nrf2*, and Δ*Nrf2*‑C without L‑theanine. Data means ± SD (n = 3 independent biological replicates, each with three technical replicates). No significant differences (*p* > 0.05), indicating *Nrf2* deletion does not affect basal physiology under the tested conditions.

**Supplementary Figure S8. Dose–response curves for L-theanine inhibition of *A. alternata mycelial* growth and spore germination**

(A) Dose–response curve for L-theanine inhibition of *A. alternata* mycelial growth. Data points represent mean ± SD of two independent biological replicates, each with four technical replicates. Concentrations tested were 0, 0.5, 1, 2, 4, and 8 μg·mL^-1^. The EC_50_ value was 1.54 ± 0.12 μg·mL^-1^ (95% CI: 1.32–1.76 μg·mL^-1^), with R² = 0.98 and Hill slope = –1.24 ± 0.15.

(B) Dose–response curve for L-theanine inhibition of *A. alternata* spore germination. Data points represent mean ± SD of three independent biological replicates. Concentrations tested were 0, 0.25, 0.5, 1, and 2 μg·mL^-1^. For each biological replicate, four microscopic fields and approximately 50 spores per field were examined. The EC_50_ value was 0.61 ± 0.05 μg·mL^-1^ (95% CI: 0.52–0.70 μg·mL^-1^), with R² = 0.97 and Hill slope = –1.18 ± 0.12.

Curves were fitted using a four-parameter logistic regression model in GraphPad Prism 8.0. Individual data points are shown. Extra sum of squares F test confirmed that the curves for WT and ΔNrf2 were significantly different (p < 0.001).

**A**

**B**

**Supplementary Texts**

**Supplementary Text S1. Detailed protocols for RNA Extraction and RT‑qPCR**

**1. RNA Isolation and Quality Control**

Mycelial samples (~100 mg) were ground in liquid nitrogen. Total RNA was extracted using 1 mL TRIzol® Reagent (Takara) per sample. Homogenates were incubated 5 min at room temperature, and phase separation with 200 μL chloroform. After centrifugation (12,000 × g, 15 min, 4 °C), the aqueous phase was transferred, and RNA precipitated with 500 μL isopropanol. The pellet was washed twice with 75% ethanol, air dried, and dissolved in 30 μL RNase‑free water. Integrity assessed by 1% agarose gel (intact 28S and 18S rRNA bands), and concentration/purity was determined with NanoDrop 2000 (A_260_/A_280_ 1.9–2.1).

**2. cDNA synthesis**

First‑strand cDNA synthesized from 2 μg total RNA using PrimeScript™ RT Reagent Kit with gDNA Eraser (Takara) following a two‑step protocol:

Step 1 (gDNA elimination): 2 μg RNA + 1 μL gDNA Eraser + 2 μL 5× gDNA Eraser Buffer, made up to 10 μL with RNase‑free water. Incubated 42 °C for 2 min.

Step 2 (RT): Add 1 μL RT Enzyme Mix I, 1 μL RT Primer Mix, 4 μL 5× PrimeScript Buffer 2, 4 μL RNase‑free water. Incubated 37 °C for 15 min, 85 °C for 5 s.

**3. Quantitative PCR (qPCR) conditions**

qPCR was performed on an ABI 7500 Fast System using SYBR® Premix Ex Taq™ II. Each 20μL reaction contained 10 μL SYBR Premix, 0.4 μL ROX Reference Dye II, 0.8 μL each primer (10 μM), 2.0 μL cDNA (1:10 diluted), and 6.0 μL ddH_2_O. Cycling conditions were 95 °C 30 s; 40 cycles of 95 °C 5 s and 60 °C 34 s; melt curve 65–95 °C. Relative expression was calculated by 2^–ΔΔCt method with *β*‑Tubulin as the reference gene. All reactions were performed with three independent biological replicates and three technical replicates.

**Supplementary Text S2. Comprehensive methodologies for MDA, SOD, CAT, GSH, and GST Assays**

**1. Malondialdehyde (MDA) Content**

Following Hodges et al. (1999). 0.5 mL supernatant mixed with 1.0 mL of 0.5% TBA in 20% TCA, boiled at 95 °C 30 min, cooled, centrifuged 10,000 × g 10 min. Absorbance read at 532 and 600 nm. MDA (nmol·g^-1^ protein) = [(A_532_ – A_600_) × Vt] / (ε × l × Vs × Cprotein) × 1000, where ε = 155 mM^-1^·cm^-1^ and Vt = 1.5 mL. Protein determined by the Bradford method.

**2. Superoxide dismutase (SOD) activity**

NBT photoreduction assay (Beauchamp and Fridovich, 1971). The reaction (3.0 mL) contained 50 mM phosphate buffer pH 7.8, 13 mM methionine, 75 μM NBT, 2 μM riboflavin, 0.1 mM EDTA, and 50 μL enzyme extract. It was exposed to fluorescent light 15 min, and absorbance was read at 560 nm. One unit = 50% inhibition of NBT reduction. SOD (U·mg^-1^ protein) = [(A_blank – A_sample) × Vt] / (A_blank × 0.5 × Vs × Cprotein).

**3. Catalase (CAT) activity**

Monitoring H_2_O_2_ decomposition at 240 nm (Beers and Sizer, 1952). The reaction (3.0 mL) contained 50 mM phosphate buffer pH 7.0, 10 mM H_2_O_2_, 50 μL enzyme extract. The decrease in absorbance was record over 3 min. One unit = 1 μmol H_2_O_2_ decomposed per min. CAT (U·mg^-1^) = [(ΔA_240_·min^-1^) × Vt × Vr × 1000] / (ε × l × Vs × Cprotein), ε = 43.6 M^-1^·cm^-1^.

**4. Glutathione (GSH) content**

DTNB recycling assay (Tietze, 1969). The reaction contained 0.1 M phosphate buffer pH 7.4, 0.6 mM DTNB, 0.2 mM NADPH, 0.5 U·mL^-1^ glutathione reductase, and 50 μL sample. The increase in absorbance at 412 nm was monitor. GSH (μmol·g^-1^) = [(A – A_0_) / (ε × l × Cprotein)] × D, ε = 13,600 L·mol^-1^·cm^-1^.

**5. Glutathione S‑transferase (GST) activity**

Habig et al. (1974). The reaction (1.0 mL) contained 0.1 M phosphate buffer pH 6.5, 1.0 mM GSH, 1.0 mM CDNB (in 1% ethanol), and 50 μL enzyme extract. The increase in absorbance at 340 nm was monitored at 30 °C over 3 min. GST (U·mg^-1^) = [(ΔA_340_·min^-1^) × V_total] / (ε × l × V_enzyme × Cprotein), ε = 9.6 mM^-1^·cm^-1^. One unit = 1 μmol GSH‑CDNB conjugate formed per min.

**Supplementary Text S3. Detailed protocols for Southern blot probe labeling, hybridization, and detection**

**1. Genomic DNA digestion and electrophoresis**

Genomic DNA (20 μg) was digested overnight with 50 U *EcoRV* (NEB) in 100 μL at 37 °C. Complete digestion was checked by loading 5 μL on 0.8% gel. Precipitated DNA resuspended in 25 μL TE and separated on 0.8% agarose in 1× TAE at 25 V for 12 h.

**2. Gel depurination, denaturation, and transfer**

The gel was sequentially soak in depurination buffer (0.25 M HCl, 15 min), denaturation buffer (1.5 M NaCl, 0.5 M NaOH, 30 min twice), and neutralization buffer (1.5 M NaCl, 0.5 M Tris‑HCl pH 7.2, 1 mM EDTA, 30 min twice). Capillary transfer to Hybond‑N+ membrane was performed using 20× SSC overnight. DNA was cross‑link at 120 mJ·cm^-2^ and bake at 80 °C for 2 h.

**3. DIG probe synthesis**

A 500 bp probe targeting upstream region of *Nrf2* generated by PCR with DIG‑11‑dUTP (Roche) at dTTP:DIG‑dUTP ratio 3:1. The probe was purified by Sephadex G‑50. Labeling efficiency was assessed by dot blot (detection limit 0.1 pg·μL^-1^).

**4. Hybridization**

The membrane pre‑hybridized in DIG Easy Hyb at 42 °C for 2 h. Denatured probe added at 25 ng·mL^-1^, and hybridized was performed at 42 °C for 16 h.

**5. Post‑hybridization washes and detection**

Low‑stringency wash: 2× SSC / 0.1% SDS, room temperature, twice 5 min. High‑stringency: 0.5× SSC / 0.1% SDS at 68 °C, twice for 15 min. Blocking was performed with 1× Blocking Solution (Roche) 1 h, followed by incubation with anti‑DIG‑AP Fab fragments (1:10,000) for 30 min. The membrane was washed with maleic acid wash buffer (0.1 M maleic acid, 0.15 M NaCl, pH 7.5, 0.3% Tween 20) three times 10 min and equilibrate in detection buffer (0.1 M Tris‑HCl, 0.1 M NaCl pH 9.5). Detection was performed with CDP‑Star, and the membrane was exposed to X-ray film for 15 min.

**Supplementary Text S4. Statistical analysis, WGCNA parameters, and raw sequencing data deposition**

**1. RNA‑Seq data deposition and accession**

Raw data were deposited in NCBI SRA under BioProject PRJNA895027 (release upon publication). Three independent biological replicates per condition were used.

**2. Bioinformatics pipeline details**

Raw reads (150 bp) were trimmed with Trimmomatic v0.39 (ILLUMINACLIP: TruSeq3‑PE.fa:2:30:10, LEADING: 3, TRAILING: 3, SLIDINGWINDOW: 4:15, MINLEN: 50). Reads were aligned to *A. alternata* ASM306616v1 using HISAT2 v2.1.0. Quantification was performed with featureCounts v2.0.0. Differential expression analysis was couducted with DESeq2 v1.30.1 (FDR adjusted *p* < 0.05, |log_2_FC| ≥ 1).

**3. WGCNA parameters**

Performed was performed using the WGCNA R package (Langfelder and Horvath, 2008) on 12 h transcriptomic data (6 samples).

Soft thresholding power = 6 (R² = 0.92)

Minimum module size = 30

Dynamic tree cut with deepSplit = 2

Module merging threshold = 0.25

Module connectivity for Nrf2 = 0.82

Co-expression correlation with ABC genes *r* = 0.91

Note: Exploratory due to limited time points. These values describe co-expression only and do not establish that Nrf2 regulates ABC transporter genes.

**4. Supplementary note on EC_50_ calculation**

Four‑parameter logistic model: Y = Bottom + (Top – Bottom) / (1 + 10^ ((LogEC_50_ – X) × HillSlope)). 95% CI provided in Supplementary Table S7. Extra sum of squares F test confirmed that WT and Δ*Nrf2* curves significantly different (*p* < 0.001).

**5. Statistical analysis and multiple testing correction**

Transcriptomic data were corrected using the Benjamini–Hochberg false discovery rate (FDR < 0.05). For hypothesis-driven pairwise comparisons, Student’s t-test (two-tailed) was used. For multiple group comparisons, one-way or two-way ANOVA followed by Tukey’s post hoc test was used. EC_50_ values were calculated using a four-parameter logistic model in GraphPad Prism 8.0, and 95% confidence intervals are reported. A *p*-value < 0.05 was considered significant.
